# Breakdown of adaptive immunotolerance induces hepatocellular carcinoma in HBsAg-tg mice

**DOI:** 10.1038/s41467-018-08096-8

**Published:** 2019-01-15

**Authors:** Lu Zong, Hui Peng, Cheng Sun, Fenglei Li, Meijuan Zheng, Yongyan Chen, Haiming Wei, Rui Sun, Zhigang Tian

**Affiliations:** 1 Division of Molecular Medicine, Hefei National Laboratory for Physical Sciences at Microscale, the CAS Key Laboratory of Innate Immunity and Chronic Disease, School of Life Sciences, University of Science and Technology of China, 230027 Hefei, Anhui China; 2 Institute of Immunology, University of Science and Technology of China, 230027 Hefei, Anhui China; 3 Clinical Laboratory, The First Affiliated Hospital of Anhui Medical University, 230022 Hefei, Anhui China

## Abstract

Hepatitis B virus (HBV) can induce chronic inflammation, cirrhosis, and eventually hepatocellular carcinoma (HCC). Despite evidence suggesting a link between adaptive immunity and HBV-related diseases in humans, the immunopathogenic mechanisms involved are seldom described. Here we show that expression of TIGIT, a promising immune checkpoint in tumor immunotherapy, increases with age on hepatic CD8^+^ T cells in HBsAg-transgenic (HBs-tg) mice whose adaptive immune system is tolerant to HBsAg. TIGIT blockade or deficiency leads to chronic hepatitis and fibrosis, along with the emergence of functional HBsAg-specific cytotoxic T lymphocytes (CTLs), suggesting adaptive immune tolerance could be broken by TIGIT blockade or deficiency. Importantly, HBsAg vaccination further induces nonresolving inflammation and HCC in a CD8^+^ T cell-dependent manner in TIGIT-blocked or -deficient HBs-tg mice. Therefore, CD8^+^ T cells play an important role in adaptive immunity-mediated tumor progression and TIGIT is critical in maintenance of liver tolerance by keeping CTLs in homeostatic balance.

## Abstract (web summary)

Chronic hepatitis B virus (HBV) infection is a risk factor for hepatocellular carcinoma (HCC) and is associated with immune tolerance to HBV. Here the authors show, in a transgenic mouse model, that rescuing T cells function via inhibition of co-inhibitory receptor TIGIT results in HCC development via supporting inflammation.

## Introduction

Chronic hepatitis B virus (HBV) infection affects more than 350 million people worldwide, despite the effective HBV vaccination among the young generation. Current antiviral treatment in the clinic is hardly effective to clear the virus^1^. Accumulating evidence has shown that chronic HBV (CHB) infection is an important risk factor for hepatocellular carcinoma (HCC)^2–4^. Virologists attribute HBV-mediated hepatocarcinogenesis to the integration of the viral DNA into the host DNA and oncoprotein regulatory X protein (HBx)^5,6^. However, it has been increasingly accepted that HBV is a non-cytopathic virus and HBV pathogenesis lies mostly in immune-mediated liver injury^7–10^, which triggers the development of HCC without viral transactivation, insertional mutagenesis, and genotoxic chemicals^11^. Despite such progress, the lack of appropriate animal models that mimic HBV-related HCC has impeded studies of immune mechanisms underlying HBV-induced HCC development.

The liver is a unique immune organ that favors the induction of immune tolerance rather than immune activation^12^. During CHB infection, virus-specific CD8^+^ T cells gradually acquire expression of numerous co-inhibitory receptors^13–16^, such as PD-1, CTLA-4, and Tim-3^17,18^. Considering the contribution of immune-mediated injury in HBV pathogenesis, co-inhibitory receptors expressed by hepatic CD8^+^ T cells are important for preventing immune-driven pathology, but also result in CTL exhaustion and thereby limit viral clearance^19,20^. Blockade of co-inhibitory receptors, such as PD-1, CTLA-4, 2B4, and Tim-3^17,21–24^, and/or activation of costimulatory signals from CD137 or OX40^25–27^, could rescue CD8^+^ T cell function during HBV infection, as evidenced by improved production of interferon (IFN)-γ and cytotoxic capacity of effector CD8^+^ T cells. On the other hand, CD8^+^ T cell response could also promote hepatic inflammatory development during acute or chronic virus infection^7^, as implied by clinical and animal studies^28–30^.

The co-inhibitory receptor T cell immunoglobulin and immune receptor tyrosine-based inhibitory motif domain (TIGIT), highly expressed on activated T cells, could inhibit T cell functions after engagement with its ligand CD155 on antigen-presenting cells^31^. Moreover, it has been demonstrated that TIGIT is a characteristic marker of exhausted CD4^+^ T^32^ and CD8^+^ T cells^33^ in tumor tissue, and enforces CD8^+^ T cell exhaustion during chronic lymphocytic choriomeningitis virus (LCMV) infection^33^. In the clinic, downregulated expression of TIGIT on both CD8^+^ T and CD4^+^ T cells were observed in hepatitis C virus (HCV) patients who were cured by direct-acting antivirals, suggesting a role for TIGIT in T cell dysfunction during HCV infection^34^. In addition, TIGIT expression on T cells correlated with disease progression induced by human immunodeficiency virus (HIV) or simian immunodeficiency virus (SIV) infection^35,36^. Nevertheless, whether TIGIT contributes to HBV-mediated immune tolerance and HBV-related HCC has not been explored.

Here, a high expression of TIGIT was found on hepatic CD8^+^ T cells of HBsAg transgenic (HBs-tg) mice, which are immunologically tolerant to HBV. TIGIT blockade or TIGIT deficiency could break CD8^+^ T cell tolerance to the viral antigen in HBs-tg mice, leading to chronic hepatitis and fibrosis. Importantly, HBsAg vaccination in combination with TIGIT blockade or TIGIT deficiency in HBs-tg mice triggered HCC development in a CD8^+^ T cell-dependent manner. Thus, this study has developed a mouse model of HBV-related HCC, providing experimental evidence supporting chronic inflammation in promoting cancer and revealing unfavorable consequences of the immune checkpoint blockade.

## Results

### TIGIT blockade or deficiency leads to chronic hepatitis

**Fig. 1:**
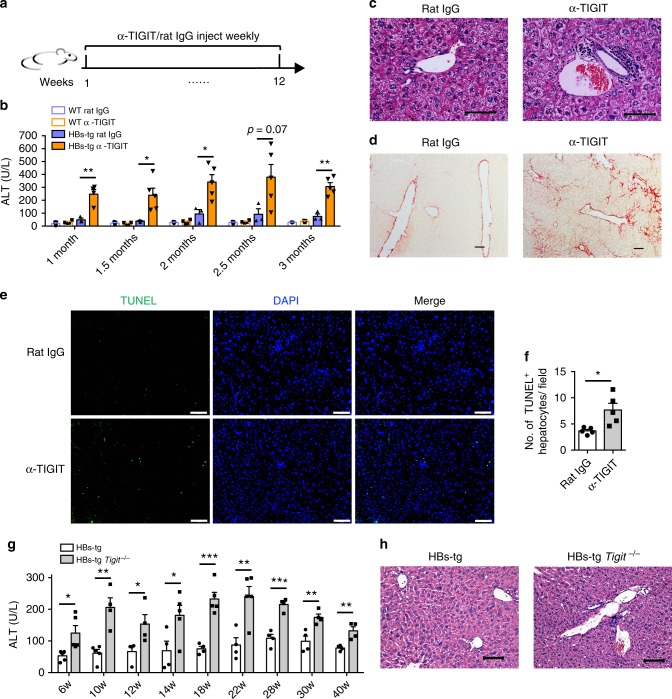
TIGIT blockade or TIGIT deficiency induces chronic hepatitis in HBs-tg mice. **a**–**e** HBs-tg or WT C57BL/6 mice were intraperitoneally injected with 200 μg α-TIGIT mAb or control rat IgG weekly. **a** Schematic of antibody treatment in mice. **b** Serum ALT levels at different time points after start of antibody treatment in HBs-tg or WT mice (*n* = 3–5 in each group). **c**, **d** Representative H&E (**c**) or sirius red staining (**d**) of liver tissue sections in HBs-tg mice after a 3-month treatment with α-TIGIT antibodies or rat IgG. Scale bar, 50 μm. **e** TUNEL labeling shows apoptotic profiles in liver tissue sections in HBs-tg mice after a 3-month treatment with α-TIGIT antibodies or rat IgG. Green-TUNEL, blue-DAPI (nuclei). Scale bar, 100 μm. **f** The number of TUNEL-positive hepatocytes per field was determined after a 3-month treatment with α-TIGIT antibodies or rat IgG. TUNEL-positive lymphocytes were excluded (*n* = 5 in each group). **g** Serum ALT levels of HBs-tg and HBs-tg *Tigit*^−/−^ mice at the indicated ages (*n* = 4–5 in each group). **h** Representative H&E staining of liver tissue sections from HBs-tg and HBs-tg *Tigit*^−/−^ mice. Scale bar, 100 μm. Statistically significant differences between the groups are presented as the mean ± SEM.: ^*^P < 0.05; ^**^P < 0.01; and ^***^P < 0.001 (two-tailed unpaired Student’s *t*-test). Data are representative of three independent experiments

It has been demonstrated that HBs-tg mice, whose hepatocytes continuously express HBV surface antigens and adaptive immune system is tolerant to HBV, can be used as a model for HBV carriers^37,38^. Given that previous studies have shown that blockade of co-inhibitory receptors could restore CD8^+^ T cell functions in HBV-carrier people^23^, we explored the consequences of blocking the TIGIT pathway in the HBs-tg mouse model. HBs-tg mice were injected weekly with anti-TIGIT monoclonal antibodies (mAb) or control rat IgG (Fig. 1a). Consistent with published reports^39^, young HBs-tg mice had normal serum alanine aminotransferase (ALT) levels compared to wild-type (WT) mice (below 50 U/L), while elder HBs-tg mice displayed slightly elevated ALT levels (Fig. 1b). We verified the blocking efficiency by flow cytometric analysis of TIGIT staining on NK cells and CD8^+^ T cells from HBs-tg mice treated with anti-TIGIT mAbs (Supplementary Fig. 1a). To further confirm that the anti-TIGIT mAb is a blocking antibody rather than depleting antibody, the absolute number of peripheral blood CD8^+^ T cells and NK cells were counted. It was found that there was no change after antibody treatment in vivo (Supplementary Fig. 1b). In addition, flow cytometric analysis with a different clone of TIGIT antibody showed that TIGIT^+^ NK cells and TIGIT^+^CD8^+^ T cells were not reduced after TIGIT blockade (Supplementary Fig. 1a), confirming that the anti-TIGIT mAb injected to mice did not induce depletion of immune cells that express TIGIT. It has been reported that, when some inhibitory pathways are ablated, there will be compensatory responses induced by increased expression of other immunosuppressive receptors^40^. To test this possibility, we examined expression of other important inhibitory receptors, such as PD-1, CTLA-4, Tim-3, KLRG-1, and Lag-3 on CD8^+^ T cells and NK cells. No changes were detected after TIGIT blockade, thus excluding the potential compensatory effects of these inhibitory receptors (Supplementary Fig. 1d). Expression of CD96, a co-inhibitory receptor sharing the same ligand CD155 with TIGIT^41^ also remained low on CD8^+^ T cells after TIGIT blockade. However, it was expressed at a lower level on NK cells in TIGIT-blocked mice compared to control mice (Supplementary Fig. 1c). The co-stimulatory molecule CD226, which competes with CD96 and TIGIT for binding to CD155^41^, maintained a high expression on CD8^+^ T cells and NK cells after TIGIT blockade (Supplementary Fig. 1c).

After weekly injections of the anti-TIGIT mAb, HBs-tg mice developed chronic hepatitis, as evidenced by gradually increased ALT levels that were significantly higher than those in rat IgG-treated HBs-tg mice (Fig. 1b). In contrast, serum ALT levels of WT mice remained in the normal range throughout the 3 months of antibody treatment. Obvious leukocyte infiltration was seen around the hepatic portal vein in liver sections of HBs-tg mice treated with the anti-TIGIT mAb (Fig. 1c), consistent with their high ALT levels. Given that chronic hepatic inflammation is associated with the development of fibrosis^42^, we analyzed the collagen deposition in the liver by sirius red staining. Severe fibrogenesis was found in HBs-tg mice after the 3-month treatment with the anti-TIGIT mAb, while the liver tissue sections from control HBs-tg mice remained normal (Fig. 1d). Moreover, TUNEL assay revealed that hepatocyte apoptosis was more obvious in anti-TIGIT-treated HBs-tg mice than in control mice (Fig. 1e, f). Since the hepatocytes in HBs-tg mice continuously express HBsAg, no difference was detected in hepatic and serum HBsAg levels between TIGIT-blockade group and the control group. This is shown by immunohistochemical staining and ELISA (Supplementary Fig. 1e, f). Moreover, serum anti-HBsAg levels in both groups were undetectable (Supplementary Fig. 1g) due to neutralizing immune reactions. In addition, serum levels of pro-inflammatory cytokines were low in TIGIT-blockade and control HBs-tg mice, and no statistical differences could be found between these groups (Supplementary Fig. 1h), suggesting that TIGIT-blockade induces local chronic inflammation in the liver of HBs-tg mice.

To further confirm the inhibition of chronic hepatitis by TIGIT, we crossed HBs-tg mice with *Tigit*^−/−^ mice to obtain HBs-tg *Tigit*^−/−^mice. Although these mice did not show significant histological evidence of inflammatory cell infiltration compared to control HBs-tg mice (Fig. 1h), dynamic examination of serum ALT levels for 10 months showed persistently higher levels of ALT in HBs-tg *Tigit*^−/−^ mice than in controls (Fig. 1g). Serum ALT levels in HBs-tg *Tigit*^−/−^ mice rose from ∼100 U/L at 6 weeks of age to 250 U/L at 6 months of age, and then fell progressively thereafter, but never returned to a point below 100 U/L. Collectively, these results revealed that the immune-tolerant HBs-tg mice develop chronic hepatitis as result of TIGIT blockade or deficiency.

### TIGIT blockade or deficiency induces HCC after vaccination

**Fig. 2:**
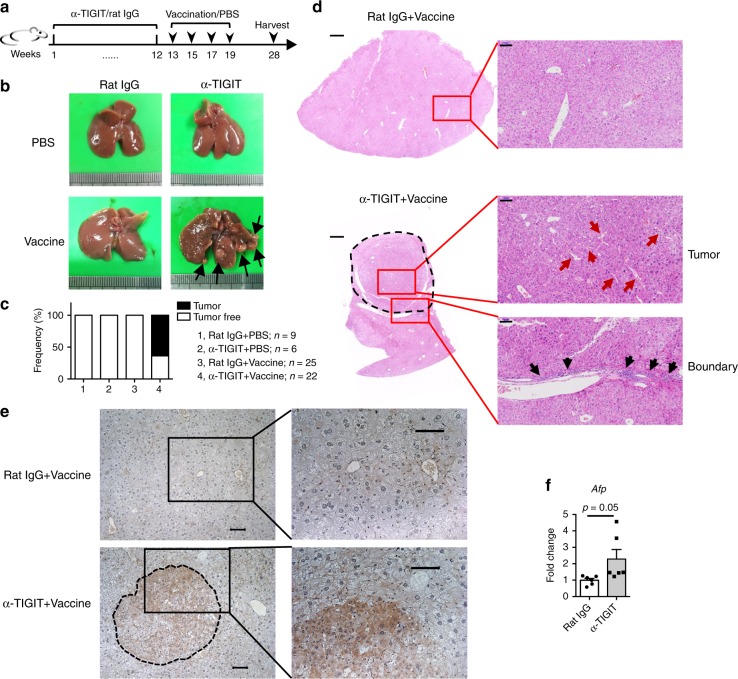
HBsAg vaccination combined with TIGIT blockade induces HCC in HBs-tg mice. **a–d** 2-month-old HBs-tg mice were intraperitoneally injected with 200 μg α-TIGIT mAb or rat IgG weekly for 3 months, then followed by intramuscular injection with 1 μg HBsAg vaccine or 50 μl PBS biweekly with four consecutive repetitions. All mice were harvested 2 months after the final vaccination. **a** Schematic of antibody treatment and vaccination in mice. **b** Representative pictures of the liver at harvest as indicated in (**a**). Black arrows indicate tumor nodules. **c** The percentage of mice with tumorigenesis. Data are pooled from three independent experiments. **d** Representative pictures of H&E staining of liver tissue sections of the mice in (**a**). The right pictures show the magnified views of the areas indicated by the squares in the left pictures (scale bars: left, 500 μm; right, 100 μm). Red arrows indicate typical trabecular HCC features. Black arrows indicate inflammatory cell infiltration. Data are representative of three independent experiments. **e** Representative images showing immunohistochemical AFP staining of paraffin-embedded liver sections from mice treated with α-TIGIT antibodies or rat IgG. The right pictures show the magnified views of the areas indicated by the squares in the left pictures. Scale bars, 50 μm. **f** Quantitative RT-PCR analysis of hepatic *Afp* gene of mice treated with rat IgG or α-TIGIT after HBsAg vaccination (*n* = 6 in each group). Statistically significant differences between the groups are presented as the mean ± SEM (two-tailed unpaired Student’s *t*-test). Data are representative of two independent experiments

To stimulate more effective immune responses within liver, HBs-tg mice were vaccinated biweekly with HBsAg four times after TIGIT blockade (Fig. 2a). Interestingly, it was found that there were tumor nodules on the liver surface in 63.64% of 9-month-old HBs-tg mice that were treated with anti-TIGIT mAb in combination with HBsAg vaccination (Fig. 2b, c). Conversely, control HBs-tg mice of the same age, which were treated with HBsAg vaccines or anti-TIGIT mAb alone, did not have liver tumor nodules. Hematoxylin and eosin (H&E) staining of tumorous liver tissues from HBs-tg mice receiving combined treatment displayed the destructive liver architecture and typical trabecular HCC features (red arrows) (Fig. 2d). Moreover, hepatocyte nuclear analysis showed prominent nucleoli around the tumor region. In the boundary zone between tumor and non-tumor tissue, there were infiltrative leukocytes surrounding the tumor region (black arrows). Immunohistochemical staining showed α-fetoprotein (AFP) was highly expressed in tumorous liver tissues (Fig. 2e). Consistent with this, quantitative PCR also showed liver tissues of HBsAg vaccinated α-TIGIT-treated mice had more *Afp* gene expression than control group (Fig. 2f). AFP is an important maker in clinic HCC diagnosis^43,44^. Therefore, HBs-tg mice displayed the classic histological features of HCC after TIGIT blockade and HBsAg vaccination.

**Fig. 3:**
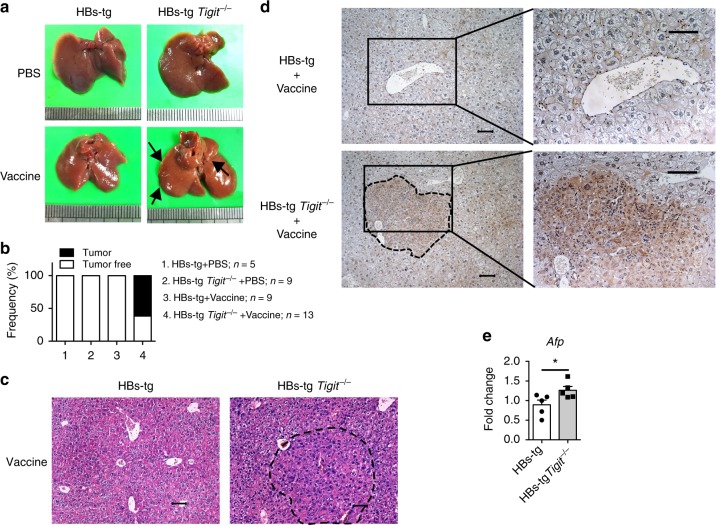
HBs-tg *Tigit*^−/−^ mice develop HCC following HBsAg vaccination. **a–c** 6-month-old HBs-tg *Tigit* ^−/−^ mice and control HBs-tg mice were intramuscularly injected with 1 μg HBsAg vaccine or 50 μl PBS biweekly with consecutive repetitions. All mice were harvested 2 months after the final vaccination. **a** Representative pictures of the liver are shown. Black arrows indicate tumor nodules. **b** The percentage of mice with tumorigenesis was calculated. Data are pooled from three independent experiments. **c** H&E staining of liver tissue sections from vaccinated HBs-tg and HBs-tg *Tigit* ^−/−^ mice. Scale bar, 50 μm. **d** Representative images showing immunohistochemical AFP staining of paraffin-embedded liver sections from vaccinated HBs-tg and HBs-tg *Tigit* ^−/−^ mice. The right pictures show the magnified views of the areas indicated by the squares in the left pictures. Scale bars, 50 μm. **e** Quantitative RT-PCR analysis of hepatic *Afp* gene of vaccinated HBs-tg and HBs-tg *Tigit* ^−/−^ mice (*n* = 5 in each group). Statistically significant differences between the groups are presented as the mean ± SEM. ^*^P < 0.05 (two-tailed unpaired Student’s *t*-test). Data are representative of two independent experiments

Consistently, 61.54% of the HBs-tg *Tigit*^−/−^ mice also developed HCC after HBsAg vaccination (Fig. 3a, b). H&E staining indicated the destruction of normal hepatocytes by the expanding tumors in these mice (Fig. 3c), and the cells in tumor regions displayed a more compact and tighter architecture compared to vaccinated HBs-tg mice. AFP-positive liver sections were seen in the tumorous liver area of vaccinated HBs-tg *Tigit*^−/−^ mice (Fig. 3d), which also had higher *Afp* gene expression than vaccinated HBs-tg mice (Fig. 3e). Therefore, these murine models collectively suggest that excessive immune responses, induced by HBsAg vaccination in combination with TIGIT blockade or deficiency, trigger the occurrence of HCC in HBs-tg mice.

### CD8^+^ T is vital in adaptive immune-induced hepatitis and HCC

**Fig. 4:**
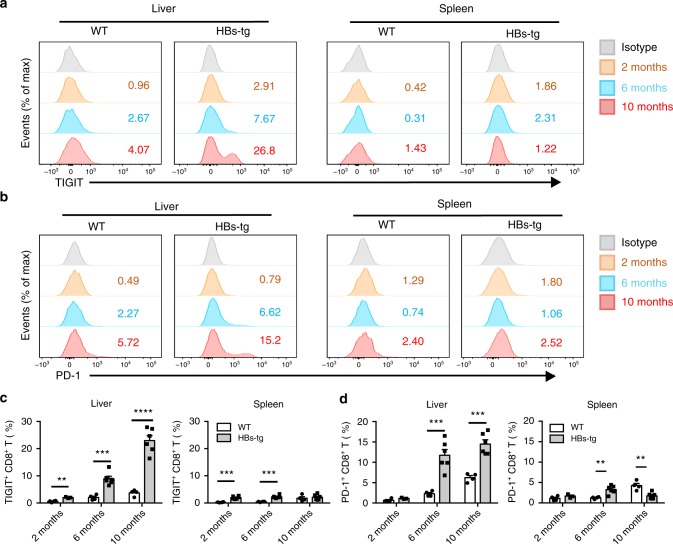
Gradually increased expression of TIGIT on CD8^+^ T cells in the liver of HBs-tg mice. **a** Representative flow cytometric graphs showing TIGIT expression on CD8^+^ T cells in the liver and spleen of 2, 6, and 10-month-old WT and HBs-tg mice. Numbers indicate the percentages of TIGIT^+^ cells among CD8^+^ T cells. **b** Representative flow cytometric graphs showing PD-1 expression on CD8^+^ T cells in the liver and spleen of 2, 6, and 10-month-old WT and HBs-tg mice. Numbers indicate the percentages of PD-1^+^ cells among CD8^+^ T cells. **c** The statistical percentages of TIGIT^+^ cells among CD8^+^ T cells in (**a**) are shown (*n* = 3–6 in each group). **d** The statistical percentages of PD-1^+^ cells among CD8^+^ T cells in (**b**) are shown (*n* = 3–6 in each group). Statistically significant differences between the groups are presented as the mean ± SEM. ^**^P < 0.01; ^***^P < 0.001; and ^****^P < 0.0001 (two-tailed unpaired Student’s *t*-test). Data are pooled from three independent experiments

Considering that dysfunction of CD8^+^ T cells correlates with HBV-induced immunotolerance, we thus examined co-inhibitory receptor expression by CD8^+^ T cells. Flow cytometric analysis revealed that TIGIT expression on hepatic CD8^+^ T cells was significantly higher than that on splenic counterparts (Fig. 4a, c), supporting the viewpoint that liver favors the induction of tolerance rather than immunity^45^. In particular, the frequency of hepatic TIGIT^+^ CD8^+^ T cells progressively increased with age in HBs-tg mice, ranging from 2% at the age of 2 months to 9% at the age of 6 months, and exceeding 20% at 10 months; this was higher than that in WT mice at the same age (Fig. 4a, c). Since PD-1 is usually expressed at high levels on CD8^+^ T cells during chronic viral infection^33^, we examined PD-1 expression as well. Consistent with the expression pattern of TIGIT, PD-1 expression on hepatic CD8^+^ T cells also increased with age, with significantly higher levels in HBs-tg mice compared to WT mice (Fig. 4b, d). These data raise the possibility that expression of these co-inhibitory receptors by CD8^+^ T cells might be associated with hepatic immune tolerance in HBs-tg mice.

**Fig. 5:**
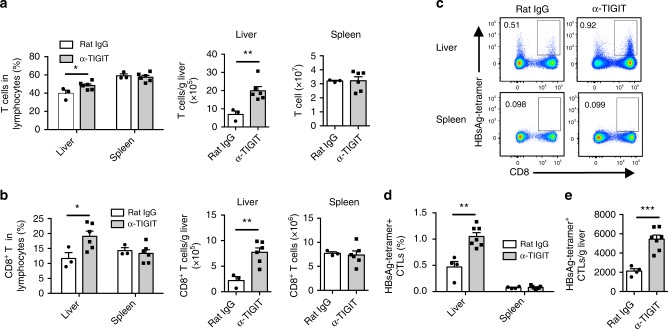
HBsAg-specific CD8^+^ T cells dramatically increase after TIGIT blockade in the liver. **a–e** HBs-tg mice were treated with rat IgG or α-TIGIT mAb for 3 months. **a**, **b** The percentage and absolute number of total T cells (**a**) and CD8^+^ T (**b**) cells in the liver and spleen of rat IgG or α-TIGIT-treated HBs-tg mice (*n* = 3, 6; Rat IgG, α-TIGIT). **c** Representative flow cytometric graphs showing HBsAg-specific CTLs in the liver and spleen of rat IgG or α-TIGIT-treated HBs-tg mice. **d** The statistical percentages in (**c**) are shown (*n* = 4, 7; Rat IgG, α-TIGIT). **e** The absolute numbers of liver HBsAg-specific CTLs are shown (*n* = 4, 7; Rat IgG, α-TIGIT). Statistically significant differences between the groups are presented as the mean ± SEM. ^*^P < 0.05; ^**^P < 0.01; and ^***^P < 0.001 (two-tailed unpaired Student’s *t*-test). Data are representative of three independent experiments

Having demonstrated TIGIT blockade-induced chronic hepatitis, we next sought to investigate whether CD8^+^ T cells break immune tolerance to HBsAg in our models. As expected, the ratio and absolute number of total T cells and CD8^+^ T cells in the liver of HBs-tg mice significantly increased after persistent blockade of TIGIT (Fig. 5a, b). Importantly, a higher proportion of HBsAg-specific cytotoxic T lymphocytes (CTLs) appeared in the liver of HBs-tg mice treated with anti-TIGIT mAb for 3 months compared to control HBs-tg mice (Fig. 5c–e and Supplementary Fig. 2a). Consistently, immunofluorescence analysis showed infiltration of CD8-positive cells in the liver tissue after TIGIT blockade (Supplementary Fig. 2d). However, no change was observed in the spleen, and liver draining lymph nodes (LNs) (Fig. 5c, d and Supplementary Fig. 2b, c).

**Fig. 6:**
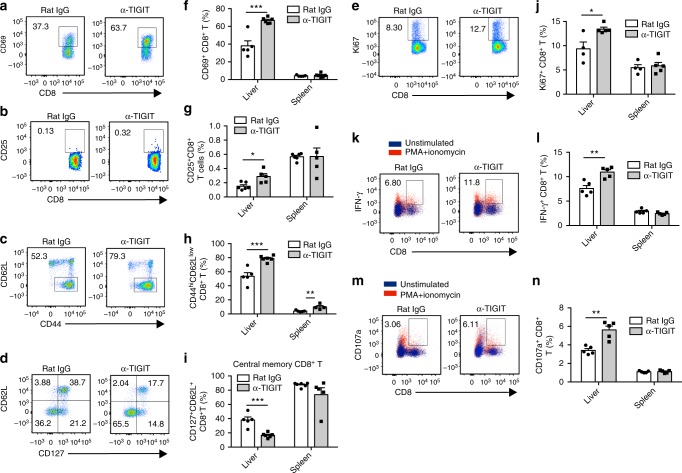
Functional tolerance of hepatic CD8^+^ T cells is broken after TIGIT blockade in HBs-tg mice. **a–n** HBs-tg mice were treated with rat IgG or α-TIGIT mAb for 3 months. **a–e** Representative flow cytometry graphs showing hepatic CD69^+^CD8^+^ T cells (**a**), CD25^+^CD8^+^ T cells (**b**), CD44^hi^CD62L^low^CD8^+^ T cells (**c**), CD127^+^CD62L^+^CD8^+^ T cells (**d**), and Ki67^+^CD8^+^ T cells (**e**) in rat IgG or α-TIGIT-treated HBs-tg mice. **f–j** Percentages of hepatic and splenic CD69^+^CD8^+^ T cells (**f**), CD25^+^CD8^+^ T cells (**g**), CD44^hi^CD62L^low^CD8^+^ T cells (**h**), CD127^+^CD62L^+^CD8^+^ T cells (**i**), and Ki67^+^CD8^+^ T cells (**j**) in rat IgG or α-TIGIT-treated HBs-tg mice (*n* = 4–6 in each group). **k–n** Expression of CD107a (**k**, **l**) and intracellular IFN-γ (**m**, **n**) by hepatic and splenic CD8^+^ T cells of mice after ex vivo stimulation with PMA and ionomycin (*n* = 5 in each group). Statistically significant differences between the groups are presented as the mean ± SEM. ^*^P <0.05; ^**^P < 0.01; and ^***^P < 0.001 (two-tailed unpaired Student’s *t*-test). Data are representative of at least two independent experiments

Moreover, it was shown that expression of CD69, a cell surface marker commonly used for quantifying lymphocyte activation^46^, increased in hepatic CD8^+^ T cells, but not in the splenic counterparts from TIGIT-blocked HBs-tg mice (Fig. 6a, f). Expression of the activation marker CD25 (Fig. 6b, g) and the proliferation marker Ki67 (Fig. 6e, j) also increased after TIGIT blockade. In addition, the frequency of CD8^+^ T cells with an effector memory phenotype (CD44^hi^CD62L^low^) in liver also dramatically increased (Fig. 6c, h). In contrast, hepatic CD8^+^ T cells with a central memory phenotype (CD127^+^CD62L^+^) significantly decreased after TIGIT blockade (Fig. 6d, i). Consistent with this trend, an increased absolute number of hepatic CD69^+^CD8^+^ T cells, CD25^+^CD8^+^ T cells, CD44^hi^CD62L^low^CD8^+^ T cells, and Ki67^+^CD8^+^ T cells was observed in TIGIT-blocked HBs-tg mice (Supplementary Fig. 3a–d), while the number of central memory CD8^+^ T cells mildly decreased (Supplementary Fig. 3e). Upon stimulation with PMA/ionomycin, hepatic CD8^+^ T cells in anti-TIGIT mAb-treated mice had higher expression of both IFN-γ (Fig. 6k, l) and CD107a (Fig. 6m, n) than those in control mice. To further evaluate the impact of TIGIT blockade on CD8^+^ T cell responses to HBV antigen, lymphocytes from HBs-tg mice were blocked by anti-TIGIT mAb in vitro and stimulated by HBsAg peptide for 4 days. We found increased expression of IFN-γ, CD107a, TNF-α, IL-2, and Ki67 in TIGIT-blocked CD8^+^ T cells (Supplementary Fig. 4a, b). These results suggest that TIGIT blockade accompanies the functional restoration of hepatic CD8^+^ T cells in HBs-tg mice.

Since previous studies have shown that TIGIT could negatively regulate NK cell function and thus protects the body from acute liver injury^47^, we wondered whether NK cells play a role in TIGIT blockade-induced chronic hepatitis. However, no significant changes in hepatic NK cell frequency and number were observed after TIGIT blockade in HBs-tg mice (Supplementary Fig. 5a). Additionally, neither IFN-γ production nor CD107a expression by NK cells were altered (Supplementary Fig. 5b, c). TIGIT blockade did not alter the frequency of regulatory T (Treg) cells (Supplementary Fig. 5d, e), which also express high levels of TIGIT^48^. These results suggest that NK cells and Treg cells may not be responsible for TIGIT blockade-induced chronic liver inflammation.

**Fig. 7:**
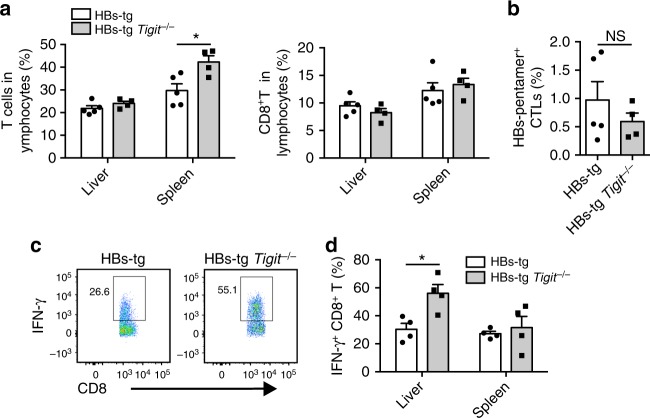
Functional tolerance of hepatic CD8^+^ T cells cannot be maintained in HBs-tg *Tigit*^−/−^ mice. **a** The percentage of total T cells and CD8^+^ T cells in the liver and spleen of HBs-tg *Tigit*^−/−^ and control HBs-tg mice (*n* = 5, 4; HBs-tg, HBs-tg *Tigit*^−/−^). **b** The percentage of hepatic HBsAg-specific CTLs (7-AAD^−^CD3^+^ NK1.1^-^ CD8β^+^ HBsAg-pentamer^+^) among CD8^+^ T cells in HBs-tg *Tigit*^−/−^ and control HBs-tg mice is shown (*n* = 5, 4; HBs-tg, HBs-tg *Tigit*^−/−^). **c** Intracellular IFN-γ staining of hepatic CD8^+^ T cells from naïve 10-month-old HBs-tg *Tigit* ^−/−^ and control HBs-tg mice in response to stimulation with PMA and ionomycin. **d** The statistical percentages in (**c**) are shown (*n* = 4 in each group). Statistically significant differences between the groups are presented as the mean ± SEM. NS, not significant; ^*^P < 0.05 (two-tailed unpaired Student’s *t*-test). Data are representative of three independent experiments

However, the frequency of total T cells and CD8^+^ T cells in the liver of HBs-tg *Tigit*^−/−^ mice was not significantly different from that of control HBs-tg mice (Fig. 7a). Likewise, TIGIT deficiency did not result in increased HBsAg-specific CTLs (Fig. 7b). Nevertheless, liver CD8^+^ T cells in HBs-tg *Tigit*^−/−^ mice displayed enhanced ability to secrete IFN-γ relative to those in control mice (Fig. 7c, d), suggesting that TIGIT deficiency in HBs-tg mice restores CD8^+^ T cell response.

**Fig. 8:**
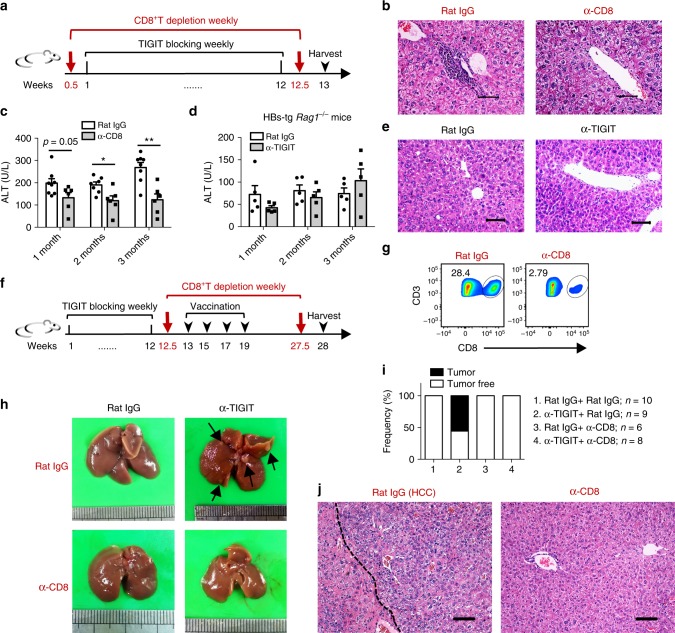
TIGIT blockade-induced chronic liver inflammation and HCC depend on CD8^+^ T cells. **a–c** HBs-tg mice were intraperitoneally injected with 100 μg CD8^+^ T cell depleting antibody (α-CD8) or rat IgG weekly for 3 months. Meanwhile, all mice were intraperitoneally injected with 200 μg α-TIGIT mAb weekly for 3 months. **a** Schematic of antibody treatment in HBs-tg mice. **b** Representative pictures of the H&E staining of liver tissue sections from mice in (**a**). Scale bar, 50 μm. **c** ALT levels of HBs-tg mice in (**a**) at different time points after start of antibody treatment (*n* = 6–8 in each group). **d**, **e** HBs-tg *Rag1*^−/−^ mice were intraperitoneally injected with 200 μg α-TIGIT mAb or rat IgG weekly. **d** ALT levels were measured at different time points after start of antibody treatment (*n* = 5 in each group). **e** Representative pictures of H&E staining of liver tissue sections from HBs-tg *Rag1*^−/−^ mice after 3 months of TIGIT blockade or rat IgG treatment. Scale bar, 100 μm. **f**–**j** HBs-tg mice were intraperitoneally injected with 200 μg α-TIGIT mAb or rat IgG weekly for 3 months, followed by weekly intraperitoneal injection with 100 μg CD8^+^ T cell depleting antibody (α-CD8) or rat IgG until harvest. At the 13th week, mice were intramuscularly injected with 1 μg HBsAg vaccine biweekly. All mice were harvested 2 months after the final vaccination. **f** Schematic of antibody treatment in HBs-tg mice. **g** Representative flow cytometry graphs showing the efficiency of CD8^+^ T cell depletion. **h** Representative pictures of the liver from HBs-tg mice. Black arrows indicate tumor nodules. **i** The percentage of mice with tumorigenesis was calculated. **j** H&E staining of liver tissue sections from HBs-tg mice. The area on the right side of the dashed line in the picture shows the tumor. Scale bars, 100 μm. Statistically significant differences between the groups are presented as the mean ± SEM. ^*^P < 0.05 and ^**^P < 0.01 (two-tailed unpaired Student’s *t*-test). Data are representative of two independent experiments

To investigate whether CD8^+^ T cells are responsible for promoting liver inflammation in these models, HBs-tg mice were depleted of CD8^+^ T cells during long-term treatment with the anti-TIGIT mAb (Fig. 8a). It was found that severe leukocyte infiltration in the liver was abolished after TIGIT blockade when CD8^+^ T cells were depleted (Fig. 8b). Simultaneously, ALT levels significantly decreased and returned to normal (Fig. 8c). To further confirm this trend, HBs-tg *Rag1*^−/−^ mice that lack the adaptive immune system were bred and injected weekly with the anti-TIGIT mAb for up to 3 months. In contrast to the observations in HBs-tg mice, TIGIT blockade failed to increase ALT levels and induce liver injury in HBs-tg *Rag1*^−/−^ mice (Fig. 8d, e), suggesting a critical role of adaptive immunity in mediating TIGIT blockade-induced chronic hepatitis.

To determine the potential roles of CD8^+^ T cells in inflammation-triggered HCC development, CD8^+^ T cells were depleted in TIGIT-blocked HBs-tg mice during the period of HBsAg vaccination (Fig. 8f, g). Notably, none of these mice had liver tumor nodules after CD8^+^ T cell depletion (Fig. 8h, i). Histological staining revealed normal liver architecture in these mice (Fig. 8j). Collectively, these data demonstrated that CD8^+^ T cells were the major players in initiating liver inflammation and promoting tumorigenesis in this HBV-related HCC mouse model.

**Fig. 9:**
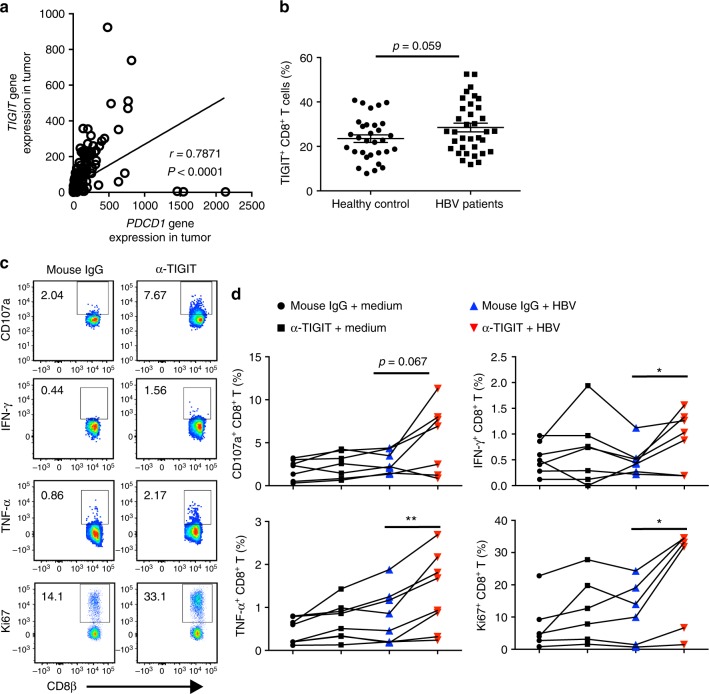
TIGIT blockade enhances antiviral responses of CD8^+^ T cells from CHB patients. **a** Correlation between the gene expressions of *TIGIT* and *PDCD1* in the HCC tumor tissue. Data are from the TCGA database. Spearman’s correlation coefficients (*r*) and *P-*values are shown. **b** TIGIT expression on peripheral blood CD8^+^ T cells in CHB patients and healthy controls was analyzed by flow cytometry (*n* = 31, 35; Healthy controls, HBV patients). Each dot represents one individual. Statistically significant differences between the groups are presented as the mean ± SEM (two-tailed unpaired Student’s *t*-test). Data are pooled from three independent experiments. **c**, **d** The peripheral blood mononuclear cells (PBMCs) from HBV patients were stimulated with HBsAg peptide in the presence of α-TIGIT or control antibody for 10 days and were then tested by intracellular cytokine staining. **c** Representative flow cytometric graphs showing expression of CD107a, IFN-γ, TNF-α, and Ki67 in CD8^+^ T cells from samples treated with mouse IgG or α-TIGIT mAb. **d** The statistical percentages in (**c**) are shown (*n* = 7, 7, 8, 6; CD107a, IFN-γ, TNF-α, Ki67). Statistically significant differences are calculated by two-tailed paired Student’s *t*-test: ^*^P < 0.05 and ^**^P < 0.01. Data are representative of three independent experiments

To investigate the clinical relevance of our findings, we collected the tumor-related data from TCGA database, and found higher *TIGIT* gene expression in intratumoral region of HCC patients than in paratumor region (Supplementary Fig. 6) and a positive correlation between *TIGIT* and *PDCD1* expression in HCC tumor tissue (Fig. 9a)^49,50^. Moreover, flow cytometric analysis revealed that CD8^+^ T cells in CHB patients expressed higher levels of TIGIT than those in healthy controls (Fig. 9b). Peripheral blood mononuclear cells (PBMCs) from CHB patients were isolated, incubated with anti-TIGIT mAb or control mouse IgG for 45 min, and then stimulated with HBsAg peptide for 10 days in vitro. We found that TIGIT blockade resulted in greater HBV-specific CD8^+^ T cell responses in CHB patients, as evidenced by increased expression of IFN-γ, CD107a, TNF-α, and Ki67 (Fig. 9c, d), suggesting that TIGIT blockade restores antiviral functions of CD8^+^ T cells in CHB patients.

## Discussion

As interest in the investigation of using immune checkpoint inhibitors in cancer therapy is continuing to grow, efforts have been made to explore the roles of co-inhibitory receptors in regulating immune responses to infections. TIGIT, a novel co-inhibitory receptor identified as a promising therapeutic target for immune modulation^51^, has been demonstrated to play a critical role in chronic viral infections, such as LCMV, HCV, SIV, and HIV infections^33–36^. However, its role in HBV infection, a global public health problem that is associated with hepatic fibrosis and HCC development, has not been reported. Our results provide evidence that TIGIT maintains hepatic immune tolerance in HBV transgenic mice. TIGIT blockade or deficiency specifically reverses hepatic CD8^+^ T cell tolerance to HBsAg and therefore results in chronic hepatitis in HBs-tg mice. This eventually leads to HCC development when combined with HBsAg vaccination. Thus, our study has established a mouse model of HBV-induced HCC suggesting that immune checkpoint therapy in HBV carriers may increase the risk of developing chronic hepatitis and liver cancer.

It is widely accepted that adaptive immunity is responsible for both viral clearance and liver damage^52,53^. For instance, CD8^+^ T cells have long been recognized as a vital player in antiviral and antitumor immune responses^54^; on the other hand, excessive CD8^+^ T cell activation leads to exaggerated inflammatory responses, and chronic inflammation can further promote tumorigenesis^7^. However, studies on the mechanisms underlying the progression of HBV infection-induced chronic hepatitis to HCC are rare, largely due to the lack of easily reproduced models. There are evidences showing that viral clearance and liver injury during acute HBV infection in chimpanzees are mediated by CD8^+^ T cells^28^. Moreover, the close association between the magnitude of virus-specific CTL responses and liver injury severity has been shown in studies in patients acutely infected with HBV^8^, and chimpanzees acutely or chronically infected with HCV^29,30^. Additionally, two independent groups have reported CD8^+^ T cell-mediated the development of HBV-related HCC in HBs-tg mice. For instance, Wang et al. found that chronic immune-mediated liver damage by continuous stimulation of the costimulatory receptor CD137 is sufficient to trigger HCC, but non-specific CD8^+^ T cells played the central role in this progression^25^. Nakamoto et al. observed that HBV-transgenic mice developed a severe necroinflammatory liver disease and HCC when their immune system was destroyed by thymectomy and irradiation and replaced by sequentially infusing nontransgenic bone marrow cells and spleen cells from HBsAg-immunized nontransgenic mice^11,55^. Compared to the animal models used in these studies, we developed a mouse model that mimics the development of HCC mediated by HBsAg-specific CTLs.

As an important inhibitory receptor, TIGIT may play dual roles in different stages of tumor development. Before tumor appearance, TIGIT maintains hepatic immune tolerance and induces CD8^+^ T cell dysfunction in HBV transgenic mice, thereby inhibiting immune-mediated injury and delaying tumor initiation. However, we observed higher *TIGIT* gene expression in intratumoral region of HCC patients than in paratumor region by TCGA database. The increased TIGIT expression might promote tumor progression by dampening effector lymphocyte-mediated anti-tumor immunity, once the tumor has finally developed^51^. Therefore, regarding the distinct roles of TIGIT in tumor initiation and progression, the use of checkpoint inhibitors at different disease stages might lead to opposing effects.

In our mouse model, it is possible that CD8^+^ T cells mediate hepatitis and HCC development through producing cytokines and direct cytotoxicity to hepatocytes. First, CD8^+^ T cells could secrete inflammatory cytokines such as IFN-γ, which can induce hepatocyte apoptosis^56,57^. Wang et al. also reported that CD8^+^ T cell-derived IFN-γ induced fibrosis-promoting cytokines/chemokines in the HBV-related HCC pathogenesis^25^. Meanwhile, HBsAg-specific CTLs could kill HBsAg-expressing hepatocytes directly via FASL^58^. Persistent hepatocyte apoptosis may cause the release of multiple apoptosis-associated proteins. Thus, the inflammatory responses could be further amplified and many other inflammatory cell types were recruited to the liver^59^. We have shown that many leukocytes infiltrated to liver after TIGIT blockade. However, only a proportion of the infiltrated cells were CD8^+^ T cells, suggesting infiltration of many other cell types. The chronic inflammation might be a crucial driving mechanism for the development of HCC in our study. Furthermore, liver has powerful regenerating capacities^60^. Massive hepatocellular death is usually accompanied by continuous cell turnover, which also accelerates the process of carcinogenesis^58^.

The kinetics of HBsAg-specific CTLs in the progression of chronic hepatitis to HCC showed that persistent TIGIT blockade induced a relatively high ratio of HBsAg-specific CTLs in the liver. However, whether antigen-specific CD8^+^ T cell accumulation in the liver is caused by their recruitment from lymphoid tissues or local expansion is unknown. Given that HBsAg-specific CTLs were not detected in the spleen or in liver-draining LNs, it is likely that they undergo expansion within the liver after HBsAg stimulation.

It should be noted that HBs-tg mice have been reported to spontaneously develop HCC from 14 to 16 months of age, with a disease prevalence of ~40%^37,38,61^. This process was ascribed to HBsAg particle retention within the endoplasmic reticulum of the hepatocytes, eventually leading to hepatocyte death^62^. The results presented here show that more than 60% of HBs-tg mice develop HCC following sequential treatment of anti-TIGIT mAb and vaccination by 9 months of age, exhibiting a higher frequency and earlier occurring time than non-treated HBs-tg mice. The endoplasmic reticulum stress remains to be examined to identify if the treatment presented here has an effect on endoplasmic reticulum stress.

In conclusion, we established a novel animal model for HBV antigen-specific CTL-mediated HCC, providing a valuable tool for further studies of chronic liver disease progression. Moreover, our findings also highlighted an important contribution of the co-inhibitory receptor TIGIT to HBV tolerance, which works as a safeguard keeping immune homeostasis to prevent chronic hepatitis and HCC initiation. Further studies on checkpoints immunotherapy of cancer should consider potential inflammation-induced carcinogenic effects.

## Methods

### Mice

HBV transgenic mice C57BL/6J-TgN (AlblHBV) 44Bri (namely HBs-tg mice), which contain the HBV genome S, pre-S, and X domains, were purchased from VITAL RIVER experiment animal company (Beijing, China), who obtained the mice from Jackson Laboratory (Bar Harbor, ME). Eight-week-old female nude BALB/c mice and male C57BL/6 mice were purchased from the Shanghai Experimental Animal Center (Shanghai, China). *Tigit*^−/−^ C57BL/6 mice were kindly provided by Bristol-Myers Squibb. *Rag1*^−/−^ C57BL/6 mice were purchased from Model Animal Research Center (Nanjing, China), who obtained the mice from Jackson Laboratory. HBs-tg *Tigit*^−/−^ mice and HBs-tg *Rag1*^−/−^ mice were generated and bred in house. All the above animals were housed in a specific pathogen-free facility and used in accordance with the regulations of animal care of University of Science and Technology of China. The study protocol was approved by Ethics Committee for Animal Care and Use at the University of Science and Technology of China (Authorization number: USTCACUC1701007, Hefei, China).

### Subjects

A total of 35 patients with CHB infection were enrolled at The First Affiliated Hospital of Anhui Medical University, Hefei, China. Diagnosis of chronic hepatitis B was based on positive HBsAg, antibody to hepatitis B core antigen (anti-HBc), and HBV-DNA. Among the patients, four patients were never treated. 14 of 35 underwent IFN-α treatment for 2–3 months; 11 of 35 underwent nucleoside analog (NUC) treatment, including entecavir treatment; 2 of 35 underwent IFN-α together with NUC treatment. Thirty-one healthy subjects uninfected with HBV were served as healthy controls. There is no difference of ALT level between patients and healthy controls, as most patients took medicine to reduce enzyme activity and protect liver. All patients and healthy controls were yellow race and were informed of the test. The detail information is in the Supplementary Tables 1 and 2. All human specimens were obtained with written informed consent and collected using a protocol approved by the Institutional Review Board of the University of Science and Technology of China (Authorization number: USTCEC201800004, Hefei, China).

### Co-inhibitory pathway blockade and cell depletion

Anti-mouse TIGIT monoclonal antibody (anti-TIGIT; clone 13G6) was generated by Absea (Beijing, China) and tested for its ability to block TIGIT-PVR interactions in vitro^47,51^. To block TIGIT in vivo, 200 μg of anti-TIGIT was intraperitoneally injected weekly. Rat IgG purified from rat serum (Weilai Bio-tech, China) was used as control. Anti-human TIGIT monoclonal antibody was generated by Absea (Beijing, China) and tested for its ability to block TIGIT–PVR interactions in vitro. Mouse IgG purified from mouse serum was used as control. To deplete CD8^+^ T cells, mice were intraperitoneally injected with 100 μg anti-CD8 (clone TIB210; purified from ascites fluid extracted from nude mice to which TIB210 was injected) weekly.

### Isolation of human PBMCs and in vitro expansion

PBMCs were isolated from heparinized blood by Ficoll density gradient centrifugation and resuspended in RPMI 1640 complete medium (10% fetal calf serum, 1% streptomycin/penicillin, 10 mM Hepes) in round 96-well plate. Each well contains 5 × 10^5^ cells in 200 μl medium. Then cells were incubated at 37 °C in the presence of 10 μg/ml anti-TIGIT or mouse IgG for 45 min. Next, cells were washed and stimulated with 10 μg/ml HBsAg peptide (ayw subtype; Hytest, Finland) for 10 days at 37 °C. At day 4, human IL-2 (Jinsili company, China) was added (20 IU/ml). At day 10, all cells were harvested and then stimulated with HBV peptide at 37 °C for 6 h. Monensin and anti-CD107a was added at the same time. After washing, cells were stained with surface marker, fixed, permeabilized, and subsequently stained with cytokine antibodies.

### Murine cell isolation

Hepatic mononuclear cells, splenocytes, and LN cells were isolated, respectively, as follows. Briefly, liver was passed through a 200-gauge mesh and collected after centrifugation. The cell pellet was then suspended in 40% percoll and layered on 70% percoll. The hepatic mononuclear cells sedimented at the interface of the two percoll gradients after centrifugation, were collected and washed twice with PBS. The spleen was passed through a 200-gauge mesh. Splenocytes were harvested after red blood cell lysis and washing by PBS. LNs were passed through a 200-gauge mesh, washed with PBS and harvested.

### Murine cells in vitro expansion

Isolated cells were resuspended in α-MEM tissue culture medium (10% fetal calf serum, 10 mM Hepes buffer, 5 × 10^-5^ M 2-ME, 1% streptomycin/penicillin) in round 96-well plate. Each well contains 5 × 10^5^ cells in 200 μl medium. Then cells were incubated at 37 °C in the presence or absence of 10 μg/ml anti-TIGIT or rat IgG for 45 min. Next, cells were washed and stimulated with 10 μg/ml HBsAg peptide (ayw subtype; Hytest, Finland) for 4 days at 37 °C. At day 4, all cells were harvested. And cells were stimulated with HBV peptide at 37 °C for 4 h. Monensin and anti-CD107a was added. After washing, cells were stained with surface marker, fixed, permeabilized, and lately stained with other cytokine antibody.

### Flow cytometry

This study used the following murine antibodies for flow cytometry. FITC-anti-CD69, PE-anti-CD107a, PE-anti-CTLA-4, PerCP-Cy5.5-anti-CD44, allophycocyanin (APC)-anti-CD25, and Brilliant Violet (BV) 510-anti-CD8 are from BD (San Diego, CA, USA). FITC-anti-CD3, FITC-anti-CD4, FITC-anti-CD8β, PE-anti-CD96, PerCP-Cy5.5-anti-IFN-γ, PE-Cy7-anti-CD3, PE-Cy7-anti-NK1.1, APC-anti-CD3, APC-anti-CD62L; APC-Cy7-anti-CD19, APC-Cy7-anti-CD3, PE/dazzle594-TIGIT (clone:1G9), BV605-anti-NK1.1, BV-421-CD127, and 7-AAD are all from BioLegend (San Diego, CA, USA). FITC-PD-1, APC-CD226; Alexa Fluor 647-anti-Foxp3, Alexa Fluor 660(AF 660) anti-TIGIT (clone: GIGD7), and AF 660-Ki67 were purchased from eBioscience (San Diego, CA, USA). For IFN-γ staining, cells were stimulated with 30 ng/ml PMA (CalBioChem), 1 μg/ml ionomycin (Sigma), and Monensin (10 μg/ml; Merck) for 4 h at 37 °C. For CD107a staining, anti-CD107a mAb was added as well. Cells were then stained with surface markers, fixed, permeabilized with a Foxp3 staining kit (eBioscience), and lately stained with anti-IFN-γ or isotype-matched control antibody following the manufacturer’s instructions. This study used the following human antibodies for flow cytometry. PerCP-Cy5.5-anti-CD3 (BioLegend), PE-CY7-CD8 (BD), BV510-CD56 (BioLegend), BV605-TIGIT (BioLegend), FITC-anti-IFN-γ(BD), PE-anti-TNF-α(BD), PE-CY7-anti-TIGIT (BioLegend), AF 647-anti-Ki67 (BD), APC-CY7-anti-IL-2 (BioLegend), and BV510-anti-CD107a (BioLegend). Data were collected using a flow cytometer (LSR II and LSRFortessa; BD) and analyzed using FlowJo 7.6 and FlowJo 10.0 software.

### Detection of HBsAg-specific CD8^+^ T cells

To evaluate HBV-specific CD8^+^ T cells, HBsAg_190–197_ (VWLSVIWM) peptide-specific H2-K^b^ pentamer (ProImmune, Oxford, UK) was used in accordance with the manufacturer’s instructions. HBsAg_190–197_ (VWLSVIWM) peptide-specific H2-K^b^ tetramer was obtained from the National Institutes of Health tetramer core facility. HBsAg-specific tetramer was used as follows: mononuclear cells were incubated with 1.2 μg/ml tetramer antibody at 4 °C for 1 h in phosphate buffer saline (PBS). The cells were then washed and stained for CD45, CD3, NK1.1, CD19, CD8β, and 7-AAD. Tetramer^+^ cells were analyzed in the CD45^+^7-AAD^-^CD3^+^NK1.1^-^CD19^-^CD8β^+^ population.

### Immune vaccination

Mice were injected intramuscularly with 1 μg HBsAg vaccine (adw subtype; BioKangtai Company) biweekly. This was conducted four times.

### Cytometric bead array (CBA) assay

IFN-γ, IL-2, IL-4, IL-6, tumor necrosis factor α (TNF-α), and IL-17A protein levels in serum were measured using CBA Th1, Th2, Th17 cytokine kit (BD) in accordance with the manufacturer’s instructions.

### Enzyme-linked immunosorbent assay (ELISA)

Serum HBsAg concentration was detected using a diagnostic kit for HBV surface antigen (Zhongshan Bio-tech, China) in accordance with the manufacturer’s instructions.

### Radioimmunoassay

Serum anti-HBs were measured using radioimmunoassay kits (North Institute of Biological Technology, China) in accordance with the manufacturer’s instructions.

### Hematoxylin and Eosin staining

For histological analysis, liver sections were fixed in 10% neutral buffered formalin and embedded in paraffin. Six micrometers of tissue sections were mounted on slides, deparaffinized, then stained with hematoxylin–eosin (H&E), and examined under light microscopy.

### Immunohistochemical staining

For the HBsAg and AFP staining, liver tissue sections of formalin-fixed and paraffin-embedded were stained with an anti-HBsAg (Abcam, Cambridge, UK) primary antibody or anti-AFP (ProteinTech, America) overnight at 4 ℃. Then sections were washed and stained with a biotinylated secondary immunoglobulin G antibody and a streptavidin–horseradish peroxidase conjugate (ZSGB-BIO) in accordance with the manufacturer’s instructions. Sections were developed by a diaminobenzidine peroxidase substrate kit (Vector, Burlingame, CA).

### TUNEL assay and immunofluorescence

The hepatocyte apoptosis was determined by TUNEL assay using a commercial kit (In Situ Cell Death Detection Kit, Roche, USA). The 3-OH ends of DNA in apoptotic cells are labeled with fluorescein conjugated dUTP by the terminal deoxynucleotidyl transferase. Briefly, liver tissue sections of formalin-fixed and paraffin-embedded were stained with TUNEL reaction mixture after membrane were ruptured. Next the sections were stained with PE-anti-CD8 (BD, America) overnight at 4 ℃. Then, the sections were stained with DAPI and lastly observed under a fluorescence microscope (Nikon Eclipse C1, Japan).

### Quantitative PCR

Total RNA was isolated from liver tissue using trizol reagent (Invitrogen, Camarillo, CA, USA). RT-PCR was performed using TB green premix *Ex Taq* (Takara, Japan) with an LightCycler 96 (Roche) according to the manufacturer’s instructions. *Afp* expression levels were normalized to the housekeeping gene *Gapdh*. The primer of *Gapdh:* forward (5′–3′) was TTCACCACCATGGAGAAGGC; reverse (5′–3′) was GGCATGGACTGTGGTCATGA. The primer of *Afp*: forward (5′–3′) was GTATTCCAACAGGAGGCTATG; reverse (5′–3′) was CATGGTCTGTAGGGCTTTG.

### Analysis of liver transaminase activity

Liver injury was assessed by serum enzyme activity of ALT using a commercially available kit (Rong Sheng, Shanghai, China) and detected by a Bio-Chemical Analyzer (Rayto, Shenzhen, China).

### Statistics

Correlations between variables were evaluated using the Spearman rank correlation test for data from humans. TIGIT gene expression levels of HCC patients were evaluated using the Mann–Whitney non-parametric statistical test. Significant differences between groups were determined using unpaired two-tailed Student’s *t*-tests unless otherwise specified; *P* < 0.05 was considered significantly different.

### Reporting summary

Further information on experimental design is available in the Nature Research Reporting Summary linked to this article.

## Supplementary information

Reporting Summary

Supplementary Information

Peer Review File

## Data Availability

The authors declare that all the data supporting the findings of this study are available within the article and its supplementary information files and from the corresponding authors upon reasonable request. A reporting summary for this Article is available as a Supplementary Information file.
