# Supplementary material for: Breakdown of adaptive immunotolerance induces hepatocellular carcinoma in HBsAg-tg mice
Source: Nat Commun. 2019 Jan 15;10:221. doi: 10.1038/s41467-018-08096-8 (PMC6333806; doi:10.1038/s41467-018-08096-8)
Supplement: Supplementary file 1 — Reporting Summary [file 41467_2018_8096_MOESM1_ESM.pdf]

nature research

Corresponding author(s): Zhigang Tian

Reporting Summary

Nature Research wishes to improve the reproducibility of the work that we publish. This form provides structure for consistency and transparency in reporting. For further information on Nature Research policies, see Authors & Referees and the Editorial Policy Checklist.

Statistical parameters

When statistical analyses are reported, confirm that the following items are present in the relevant location (e.g. figure legend, table legend, main text, or Methods section).

n/a	Confirmed	
☐	☒	The exact sample size (*n*) for each experimental group/condition, given as a discrete number and unit of measurement
☐	☒	An indication of whether measurements were taken from distinct samples or whether the same sample was measured repeatedly
☐	☒	The statistical test(s) used AND whether they are one- or two-sided<br>*Only common tests should be described solely by name; describe more complex techniques in the Methods section.*
☐	☒	A description of all covariates tested
☐	☒	A description of any assumptions or corrections, such as tests of normality and adjustment for multiple comparisons
☐	☒	A full description of the statistics including central tendency (e.g. means) or other basic estimates (e.g. regression coefficient) AND variation (e.g. standard deviation) or associated estimates of uncertainty (e.g. confidence intervals)
☐	☒	For null hypothesis testing, the test statistic (e.g. *F*, *t*, *r*) with confidence intervals, effect sizes, degrees of freedom and *P* value noted<br>*Give P values as exact values whenever suitable.*
☒	☐	For Bayesian analysis, information on the choice of priors and Markov chain Monte Carlo settings
☒	☐	For hierarchical and complex designs, identification of the appropriate level for tests and full reporting of outcomes
☒	☐	Estimates of effect sizes (e.g. Cohen's *d*, Pearson's *r*), indicating how they were calculated
☐	☒	Clearly defined error bars<br>*State explicitly what error bars represent (e.g. SD, SE, CI)*

Our web collection on statistics for biologists may be useful.

Software and code

Policy information about availability of computer code

Data collection	N.A.
Data analysis	Graphpad Prism 6.0 (Graphpad); FlowJo software (Tree Star,version7.6.2 or 10).

For manuscripts utilizing custom algorithms or software that are central to the research but not yet described in published literature, software must be made available to editors/reviewers upon request. We strongly encourage code deposition in a community repository (e.g. GitHub). See the Nature Research guidelines for submitting code & software for further information.

Data

Policy information about availability of data

All manuscripts must include a data availability statement. This statement should provide the following information, where applicable:

- Accession codes, unique identifiers, or web links for publicly available datasets
- A list of figures that have associated raw data
- A description of any restrictions on data availability

All data of this study are available within the article and its Supplementary Information files or from the corresponding author on reasonable request.

Field-specific reporting

Please select the best fit for your research. If you are not sure, read the appropriate sections before making your selection.

☒ Life sciences ☐ Behavioural & social sciences ☐ Ecological, evolutionary & environmental sciences

For a reference copy of the document with all sections, see nature.com/authors/policies/ReportingSummary-flat.pdf

Life sciences study design

All studies must disclose on these points even when the disclosure is negative.

Sample size	Sample size was estimated on the basis of similar research reported in the literature. In most of the experiments, 3-7 mice/group was sufficient to identify differences. In tumor experiments, 6-25 mice/group was used to identify differences. For human experiments, 31-35 people/group.
Data exclusions	In fig9.a, we excluded the highest TIGIT expression dot in both healthy control group and HBV patients group (each group exclude one dot: 48.1 for healthy control and 68.9 for HBV patients).
Replication	All data was successfully replicated at least two or three times as indicated in the figure legends.
Randomization	Mice were allocated into experimental groups randomly.
Blinding	The investigators were not blinded to group allocation during experiments. Conclusions were made based on independent experiments, quantitative parameters and statistical significance of the data.

Reporting for specific materials, systems and methods

Materials & experimental systems

n/a	Involved in the study	
☒	☐	Unique biological materials
☐	☒	Antibodies
☐	☒	Eukaryotic cell lines
☒	☐	Palaeontology
☐	☒	Animals and other organisms
☐	☒	Human research participants

Methods

n/a	Involved in the study	
☒	☐	ChIP-seq
☐	☒	Flow cytometry
☐	☒	MRI-based neuroimaging

Antibodies

Antibodies used	This study used the following murine antibodies for flow cytometry. FITC-anti-CD69, PE-anti-CD107a, PE-anti-CTLA-4, PerCP-Cy5.5-anti-CD44, allophycocyanin (APC)-anti-CD25 and Brilliant Violet (BV) 510-anti-CD8 are from BD (San Diego, CA, USA). FITC-anti-CD3, FITC-anti-CD4, FITC-anti-CD8β, PE-anti-CD96, PerCP-Cy5.5-anti-IFN-γ, PE-Cy7-anti-CD3, PE-Cy7-anti-NK1.1, APC-anti-CD3, APC-anti-CD62L; APC-Cy7-anti-CD19, APC-Cy7-anti-CD3, PE/dazzle594-TIGIT (clone:1G9), BV605-anti-NK1.1, BV-421-CD127, and 7-AAD are all from BioLegend (San Diego, CA, USA). FITC-PD-1, APC-CD226; Alexa Fluor 647-anti-Foxp3, Alexa Fluor 660(AF 660) anti-TIGIT (clone: GIGD7) and AF 660-Ki67 were purchased from eBioscienc (San Diego, CA, USA). This study used the following human antibodies for flow cytometry. PerCP-Cy5.5-anti-CD3 (BioLegend), PE-CY7-CD8 (BD), BV510-CD56 (BioLegend), BV605-TIGIT (BioLegend), FITC-anti-IFN-γ(BD), PE-anti-TNF-α(BD), PE-CY7-anti-TIGIT (BioLegend), AF 647-anti-Ki67 (BD), APC-CY7-anti-IL-2 (BioLegend), and BV510-anti-CD107a (BioLegend)
Validation	All antibodies are from commercial sources and their validation data are available on the manufacturer's website.

Eukaryotic cell lines

Policy information about cell lines

Cell line source(s)	Hybridoma cells TIB210 (anti-CD8+T, in-vivo depletion) were purchased from ATCC. Hybridoma cells of anti-mouse TIGIT monoclonal antibody and anti-human TIGIT were generated in mice by our lab.
Authentication	Authentication was performed by ATCC for TIB210 cell line (Method: STR profiling).Authentication was performed by our lab

Authentication	for hybridoma cells of anti-mouse TIGIT and anti-human TIGIT.
Mycoplasma contamination	All cell lines tested negative for mycoplasma contamination.
Commonly misidentified lines (See ICLAC register)	None.

Animals and other organisms

Policy information about studies involving animals; ARRIVE guidelines recommended for reporting animal research

Laboratory animals	HBV transgenic mice<br>C57BL/6J-TgN (AlblHBV) 44Bri (namely HBs-tg mice), which contain the HBV genome S, pre-S, and X domains, were purchased from VITAL RIVER experiment animal company (Beijing, China), who obtained the mice from Jackson Laboratory (Bar Harbor, ME). Eight-week-old female nude BALB/c mice and C57BL/6 mice were purchased from the Shanghai Experimental Animal Center (Shanghai, China). Tigit-/- C57BL/6 mice were kindly provided by Bristol-Myers Squibb. Rag1-/- C57BL/6 mice were purchased from Model Animal Research Center (Nanjing, China), who obtained the mice from Jackson Laboratory. HBs-tg Tigit-/- mice and HBs-tg Rag1-/- mice were generated and bred in house.
Wild animals	None.
Field-collected samples	None.

Human research participants

Policy information about studies involving human research participants

Population characteristics	A total of 35 patients with chronic HBV infection were enrolled at the The First Affiliated Hospital of Anhui Medical University, Hefei, China. Diagnosis of chronic hepatitis B was based on positive HBsAg, antibody to hepatitis B core antigen (anti-HBc), and HBV-DNA. Among the patients, 4 patients were never treated. 14 of 35 underwent interferon (IFN)-α treatment for 2-3 months; 11 of 35 underwent nucleoside analog (NUC) treatment, including entecavir treatment; 2 of 35 underwent IFN-α together with NUC treatment. 31 healthy subjects uninfected with HBV were served as healthy controls. There is no difference of ALT level between patients and healthy controls, as most patients took medicine to reduce enzyme activity and protect liver. All patients and healthy controls were yellow race and were informed of the test.
Recruitment	HBV patients and healthy controls were enrolled at the The First Affiliated Hospital of Anhui Medical University, Hefei, China.

Flow Cytometry

Plots

Confirm that:

☒ The axis labels state the marker and fluorochrome used (e.g. CD4-FITC).

☒ The axis scales are clearly visible. Include numbers along axes only for bottom left plot of group (a 'group' is an analysis of identical markers).

☒ All plots are contour plots with outliers or pseudocolor plots.

☒ A numerical value for number of cells or percentage (with statistics) is provided.

Methodology

Sample preparation	Hepatic mononuclear cells, splenocytes and lymph node(LN) cells were isolated respectively as previously described 64. Briefly, liver was passed through a 200-gauge mesh and collected after centrifugation. The cell pellet was then suspended in 40% percoll and layered on 70% percoll. The hepatic mononuclear cells sedimented at the interface of the two percoll gradients after centrifugation, were collected and washed twice with PBS. The spleen was passed through a 200-gauge mesh. Splenocytes were harvested after red blood cell lysis and washing by PBS. LNs were passed through a 200-gauge mesh, washed with PBS and harvested.
Instrument	Cells were analyzed using a BD LSR II or BD LSRFortessa (BD Biosciences, San Jose, CA, U.S.A.).
Software	FlowJo software (TreeStar, Ashland, OR, U.S.A.).
Cell population abundance	None.
Gating strategy	Gating strategies were indicated in Supplementary Fig 2a.

☒ Tick this box to confirm that a figure exemplifying the gating strategy is provided in the Supplementary Information.

Magnetic resonance imaging

Experimental design

Design type	N.A.
Design specifications	N.A.
Behavioral performance measures	N.A.

Acquisition

Imaging type(s)	N.A.
Field strength	N.A.
Sequence & imaging parameters	N.A.
Area of acquisition	N.A.

Diffusion MRI ☐ Used ☐ Not used

Preprocessing

Preprocessing software	N.A.
Normalization	N.A.
Normalization template	N.A.
Noise and artifact removal	N.A.
Volume censoring	N.A.

Statistical modeling & inference

Model type and settings	N.A.
Effect(s) tested	*Define precise effect in terms of the task or stimulus conditions instead of psychological concepts and indicate whether ANOVA or factorial designs were used.*

Specify type of analysis: ☐ Whole brain ☐ ROI-based ☐ Both

Statistic type for inference (See Eklund et al. 2016)	N.A.
Correction	N.A.

Models & analysis

n/a	Involved in the study
☐	☐ Functional and/or effective connectivity
☐	☐ Graph analysis
☐	☐ Multivariate modeling or predictive analysis

Functional and/or effective connectivity	N.A.
Graph analysis	N.A.
Multivariate modeling and predictive analysis	N.A.
