# Supplementary material for: Breakdown of adaptive immunotolerance induces hepatocellular carcinoma in HBsAg-tg mice
Source: Nat Commun. 2019 Jan 15;10:221. doi: 10.1038/s41467-018-08096-8 (PMC6333806; doi:10.1038/s41467-018-08096-8)
Supplement: Supplementary file 2 — Supplementary Information [file 41467_2018_8096_MOESM2_ESM.pdf]

Supplementary Information

Breakdown of adaptive immunotolerance induces hepatocellular carcinoma in HBsAg-tg mice

Lu Zong[1,2], Hui Peng[1,2], Cheng Sun[1,2], Fenglei Li[1,2], Meijuan Zheng[3], Yongyan Chen[1,2], Haiming Wei[1,2], Rui Sun[1,2], Zhigang Tian[1,2*]

[1]Division of Molecular Medicine, Hefei National Laboratory for Physical Sciences at Microscale, the CAS Key Laboratory of Innate Immunity and Chronic Disease, School of Life Sciences, University of Science and Technology of China, Hefei, Anhui 230027, China
[2]Institute of Immunology, University of Science and Technology of China, Hefei, Anhui 230027, China
[3]Clinical Laboratory, The First Affiliated Hospital of Anhui Medical University, Hefei, Anhui 230022, China

[*]To whom correspondence may be addressed. School of Life Sciences, University of Science and Technology of China, 443 Huang-Shan Road, Hefei, Anhui 230027, China. E-mail addresses: tzg@ustc.edu.cn.

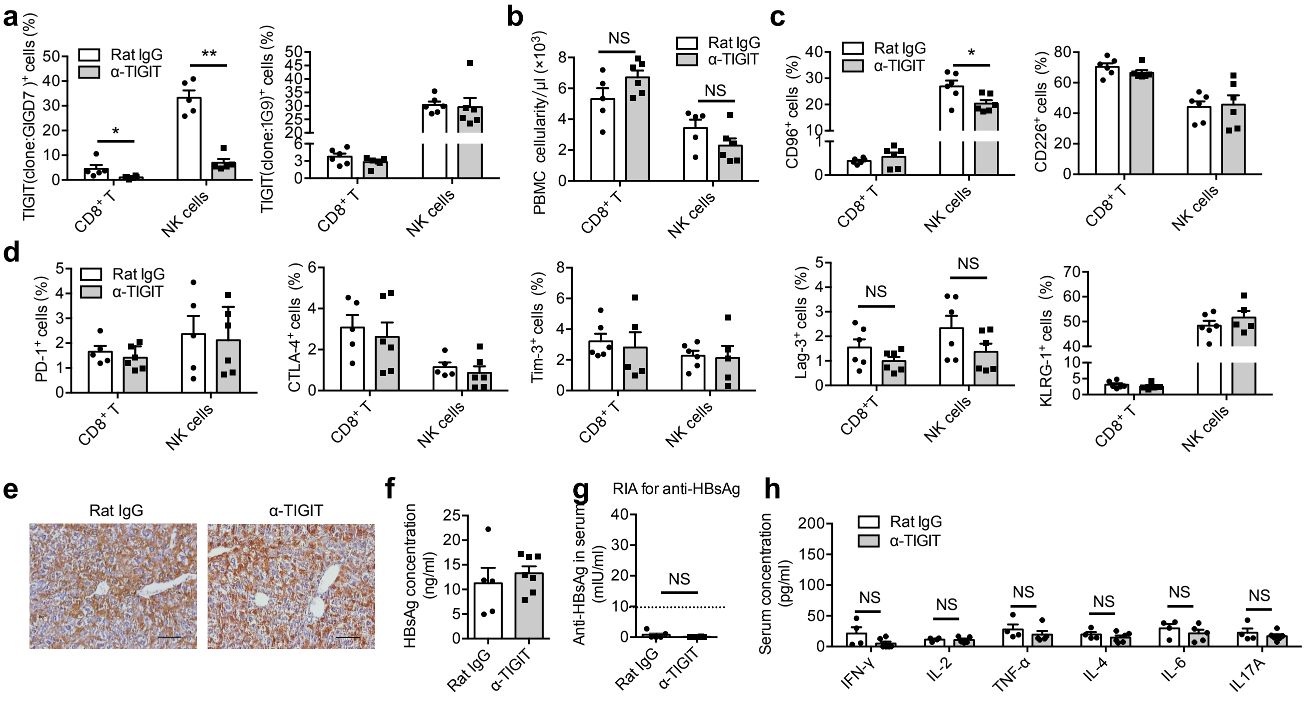
a
b
c
d
e
f
g
h
Rat IgG
α-TIGIT
TIGIT(clone:GIGD7)+ cells (%)
TIGIT(clone:1G9)+ cells (%)
CD8+ T
NK cells
PBMC cellularity/μl (×10³)
NS
CD96+ cells (%)
CD226+ cells (%)
PD-1+ cells (%)
CTLA-4+ cells (%)
Tim-3+ cells (%)
Lag-3+ cells (%)
KLRG-1+ cells (%)
HBsAg concentration (ng/ml)
RIA for anti-HBsAg
Anti-HBsAg in serum (mIU/ml)
Serum concentration (pg/ml)
IFN-γ
IL-2
TNF-α
IL-4
IL-6
IL17A

Supplementary Figure 1. TIGIT-blockade does not influence systemic immunity of HBs-tg mice. (**a-h**) HBs-tg mice were intraperitoneally injected with 200 μg α-TIGIT mAb (clone 13G6) or control rat IgG weekly. (**a**) TIGIT expression on peripheral blood $CD8^+$ T cells or NK cells from HBs-tg mice treated with α-TIGIT mAb or Rat IgG for 1.5 months was examined by flow cytometry with different clones of TIGIT antibodies (clone GIGD7 or 1G9) (n=5-6 in each group). (**b**) Total peripheral blood mononuclear cells (PBMCs) per μl isolated from HBs-tg mice treated with α-TIGIT mAb or rat IgG for 1.5 months (n=5,6; Rat IgG, α-TIGIT). (**c, d**) Expression of the indicated molecules on peripheral blood $CD8^+$ T cells or NK cells from mice treated with α-TIGIT mAb or rat IgG for 1.5 months was examined by flow cytometry (n=5-6 in each group). (**e**) Representative immunohistochemical staining of liver tissue sections for HBsAg in HBs-tg mice treated with α-TIGIT mAb or rat IgG for 3 months. Scale bars represent 50 μm. (**f**) Serum HBsAg levels were quantified by ELISA after 3-months of injections with α-TIGIT mAb or Rat IgG (n=5,7; Rat IgG, α-TIGIT). (**g**) Serum anti-HBsAg levels in HBs-tg mice treated with α-TIGIT mAb or rat IgG for 3 months were monitored by radioimmunoassay. The dashed line represents threshold value (n=5,6; Rat IgG, α-TIGIT). (**h**) Serum levels of IFN-γ, IL-2, TNF-α, IL-4, IL-6 and IL-17A in HBs-tg mice treated with α-TIGIT mAb or rat IgG for 3 months were monitored by the cytometric bead array (CBA) assay (n=4-6 in each group). Statistically significant differences between the groups are presented as the mean ± SEM.: $*P < 0.05$; $**P < 0.01$ (two-tailed unpaired Student's *t*-test). Data are representative of three independent experiments.

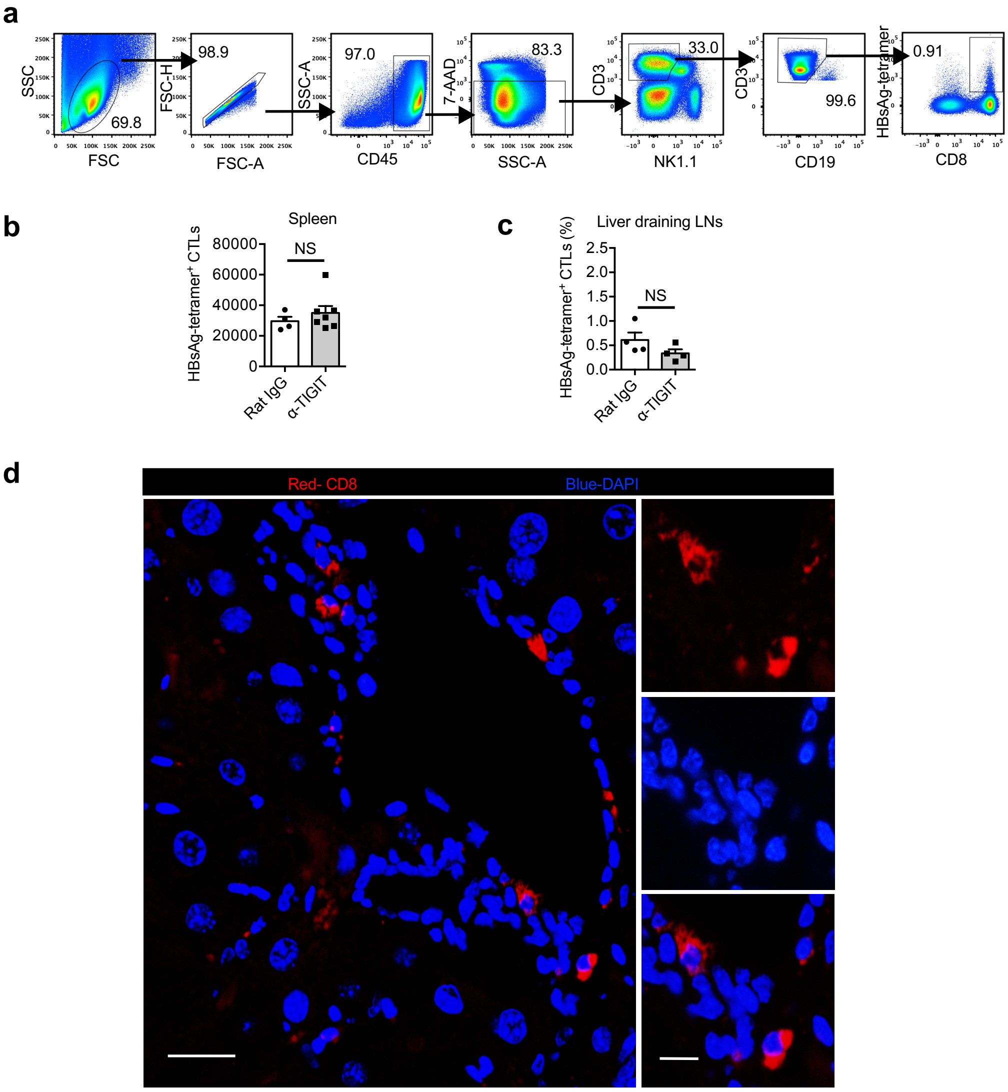

Supplementary Figure 2, related to Fig. 5. Gating strategy for HBsAg-specific CTLs. (**a**) Flow cytometry gating strategy for HBsAg-specific CTLs in the liver and spleen of HBs-tg mice after TIGIT blockade. HBsAg-specific CTLs were gated as $CD45^{+}7\text{-}AAD^{-}CD3^{+}NK1.1^{-}CD19^{-}CD8\beta^{+}$ $HBsAg\text{-}tetramer^{+}$ cells. (**b**) The absolute number of splenic HBsAg-specific CTLs in HBs-tg mice treated with α-TIGIT mAb or Rat IgG for 3 months is shown (n=4,7; Rat IgG, α-TIGIT). (**c**) The percentage of HBsAg-specific CTLs in liver draining LNs of HBs-tg mice treated with α-TIGIT mAb or rat IgG for 3 months is shown (n=4 in each group). (**d**) Immunofluorescence shows CD8 positive cells in liver tissue sections in HBs-tg mice after a 3-month treatment with α-TIGIT antibodies. Red-CD8, blue-DAPI (nuclei). Scale bar, 25μm(left), 10μm(right). Statistically significant differences between the groups are presented as the mean ± SEM. (two-tailed unpaired Student's *t*-test). Data are representative of three independent experiments.

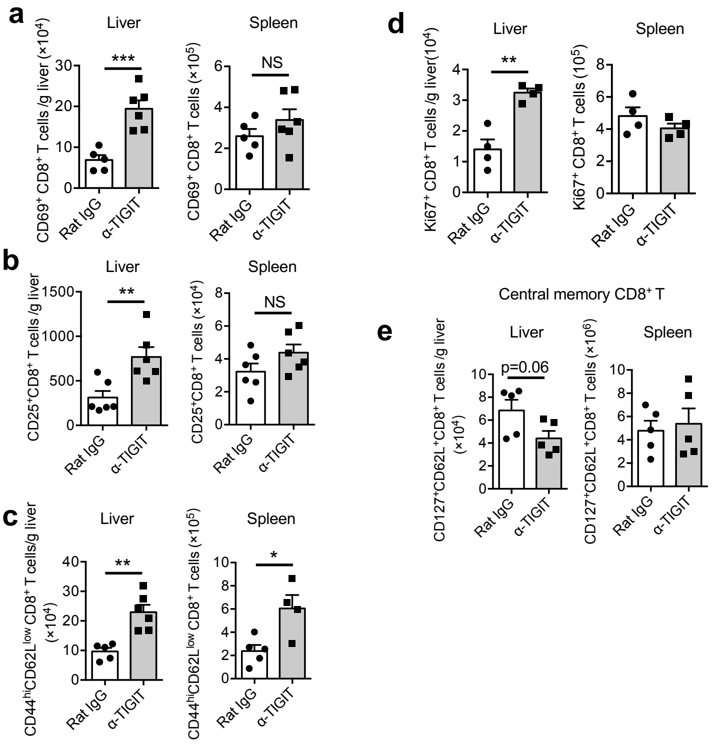

Supplementary Figure 3, related to Fig. 6. (**a-e**) HBs-tg mice were treated with rat IgG or α-TIGIT mAb for 3 months. The absolute number of total $CD69^{+}CD8^{+}$ T cells (**a**), $CD25^{+}CD8^{+}$ T cells (**b**), $CD44^{hi}CD62L^{low}$ $CD8^{+}$ T cells (**c**), $Ki67^{+}CD8^{+}$ T cells (**d**), and $CD127^{+}CD62L^{low}$ $CD8^{+}$ T cells (**e**) in the liver and spleen of Rat IgG or α-TIGIT-treated HBs-tg mice (n=4-6 in each group). Statistically significant differences between the groups are presented as the mean ± SEM.: $^{**}P < 0.01$; $^{***}P < 0.001$ (two-tailed unpaired Student's *t*-test). Data are representative of two independent experiments.

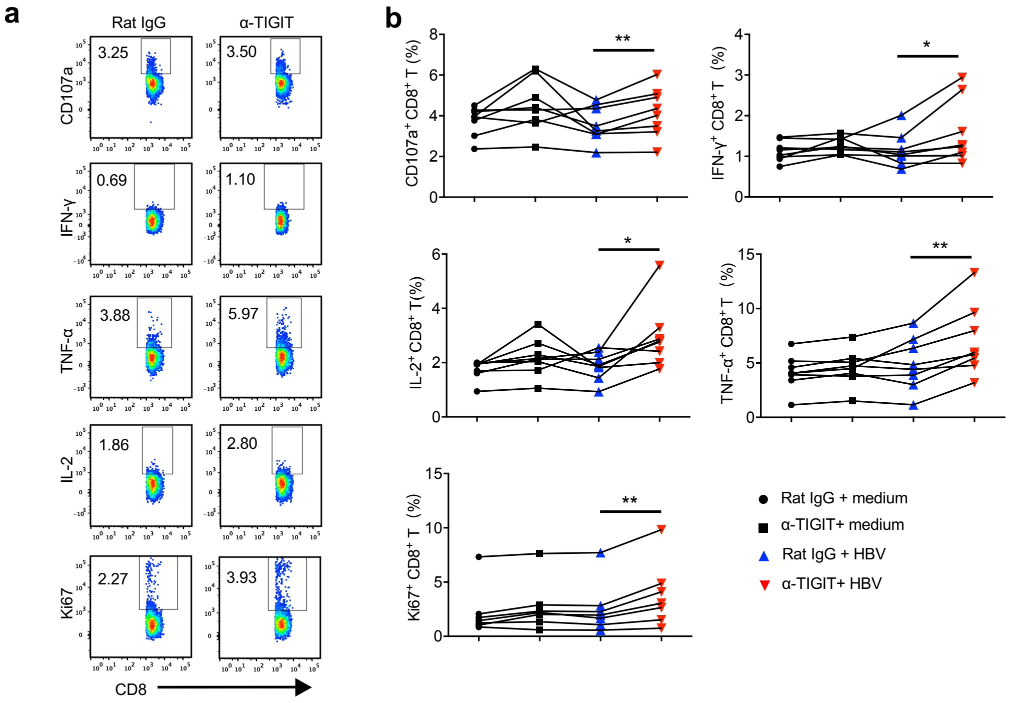

Supplementary Figure 4. Cytokine production and ki67 staining by CD8⁺ T cells after *in vitro* expansion in response to HBV peptide stimulation. Lymphocytes isolated from spleen of HBs-tg mice were expanded for 4 days by stimulation with HBsAg peptide. Cytokine production was tested by intracellular cytokine staining. (**a**) Representative flow cytometry graphs showing $CD107a^{+}CD8^{+}$ T cells, IFN-$\gamma^{+}CD8^{+}$ T cells, TNF-$\alpha^{+}CD8^{+}$ T cells, IL-$2^{+}CD8^{+}$ T cells, and $Ki67^{+}CD8^{+}$ T cells in samples treated with rat IgG or α-TIGIT mAb from the same mouse. (**b**) The statistical percentages in (**a**) are shown (n=8,8,8,8,7; CD107a, IFN-γ, IL-2, TNF-α, Ki67). Statistically significant differences are calculated by two-tailed paired T test: $*P < 0.05$, $**P < 0.01$. Data are representative of two independent experiments.

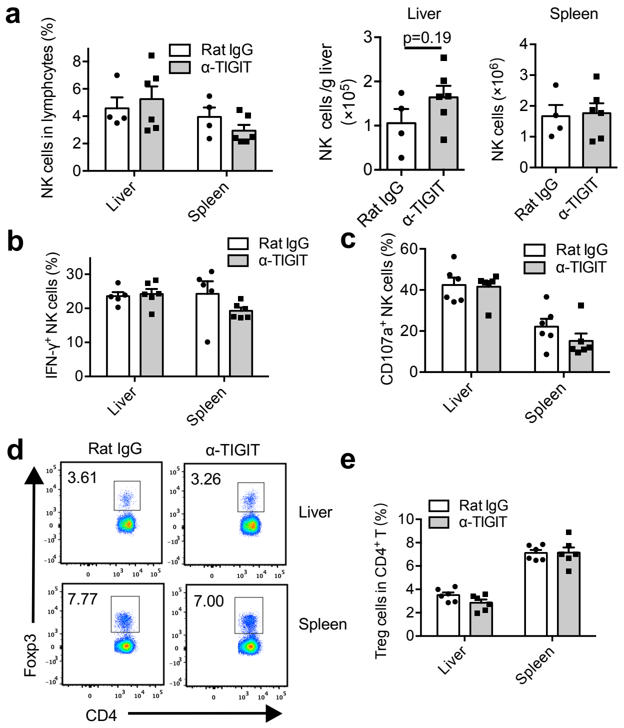

Supplementary Figure 5. NK cells and Treg cells are not influenced by TIGIT blockade in HBs-tg mice. (**a**) The percentage and absolute number of hepatic and splenic NK cells (NK1.1$^+$CD3$^-$) from HBs-tg mice treated with rat IgG or αTIGIT for 3 months (n=4,6; Rat IgG, α-TIGIT). (**b, c**) Expression of intracellular IFN-γ (**b**) (n=5,6; Rat IgG, α-TIGIT) and CD107a (**c**) (n=6 in each group). by hepatic and splenic NK cells from HBs-tg mice treated with rat IgG or α-TIGIT for 3 months in response to PMA and ionomycin stimulation. (**d, e**) Representative flow cytometry graphs (**d**) and the percentage (**e**) of CD3$^+$NK1.1$^-$CD4$^+$Foxp3$^+$ Treg cells in the liver and spleen of HBs-tg mice treated with rat IgG or α-TIGIT for 3 months (n=6 in each group). Statistically significant differences between the groups are presented as the mean ± SEM.(two-tailed unpaired Student's *t*-test). Data are representative of three independent experiments.

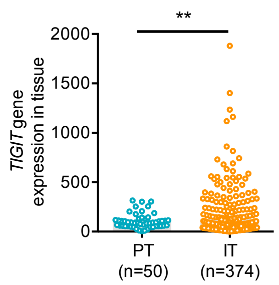

Supplementary Figure 6, *TIGIT* gene expression of intratumoral tissue was higher than paratumor tissue. (**a**) *TIGIT* gene expression levels in intratumoral (IT) tissue (n=374) and paratumor (PT) tissue (n=50) of HCC patients from the TCGA database. Each dot represents an individual patient. The results are expressed as the mean ± SEM.: **$P < 0.01$(Mann-Whitney non-parametric statistical test).

Supplementary Table 1: Characteristics of the HBV patients enrolled in the study.

**HBV patients**	**Gender**	**Age**	**ALT(U/L)**	**Therapy**
**1**	F	67	18	NUC
**2**	F	33	50	IFN-α
**3**	M	33	342	not treated
**4**	F	20	18	IFN-α
**5**	F	25	40	ETV
**6**	M	43	31	NUC
**7**	M	70	33	n/a
**8**	F	48	23	IFN-α
**9**	F	45	19	NUC
**10**	F	24	430	not treated
**11**	M	33	n/a	n/a
**12**	M	47	n/a	n/a
**13**	M	42	n/a	n/a
**14**	M	43	30	IFN-α
**15**	M	51	n/a	NUC
**16**	M	57	n/a	IFN-α
**17**	F	43	n/a	NUC
**18**	M	49	n/a	ETV
**19**	M	31	n/a	not treated
**20**	M	39	n/a	IFN-α
**21**	M	34	n/a	IFN-α
**22**	M	54	n/a	IFN-α
**23**	F	28	n/a	not treated
**24**	F	45	19	not treated
**25**	F	35	n/a	not treated
**26**	M	47	n/a	IFN-α+NUC
**27**	M	40	n/a	IFN-α
**28**	M	39	n/a	NUC
**29**	M	52	n/a	IFN-α
**30**	M	45	n/a	NUC
**31**	F	53	n/a	IFN-α+NUC
**32**	F	40	n/a	ETV
**33**	F	42	n/a	IFN-α
**34**	M	38	33	IFN-α
**35**	M	51	23	NUC

Abbreviations: n/a: not applicable; ETV: entecavir; NUC: nucleoside analog.

Supplementary Table 2: Characteristics of the healthy controls enrolled in the study.

**Healthy control**	**Gender**	**Age**	**ALT(U/L)**
**1**	M	26	17
**2**	M	29	15
**3**	M	22	34
**4**	F	22	8
**5**	M	56	23
**6**	M	55	16
**7**	F	29	12
**8**	M	56	33
**9**	M	37	46
**10**	F	36	18
**11**	F	59	11
**12**	M	47	22
**13**	M	54	23
**14**	F	44	19
**15**	M	32	38
**16**	M	47	16
**17**	F	31	14
**18**	F	30	13
**19**	M	51	23
**20**	F	52	25
**21**	F	46	13
**22**	F	50	11
**23**	M	51	28
**24**	M	31	33
**25**	M	45	64
**26**	M	43	30
**27**	F	24	17
**28**	F	45	15
**29**	M	29	43
**30**	M	37	21
**31**	F	32	20
