# Supplementary material for: Breakdown of adaptive immunotolerance induces hepatocellular carcinoma in HBsAg-tg mice
Source: Nat Commun. 2019 Jan 15;10:221. doi: 10.1038/s41467-018-08096-8 (PMC6333806; doi:10.1038/s41467-018-08096-8)
Supplement: Supplementary file 3 — Peer Review File [file 41467_2018_8096_MOESM3_ESM.pdf]

Reviewers' Comments:

Reviewer #1:
Remarks to the Author:
In this manuscript, Dr. Tian and colleagues have observed that TIGIT-blockade or deficiency induces chronic hepatitis and fibrosis in HBs-tg mice. HBsAg vaccination further induced HCC. CD8+ T cell depletion abolishes the development of hepatitis or HCC. This study established a novel animal model for HBV antigen-specific CTL-mediated HCC, based on TIGIT blockade and vaccination in HBs-tg mice. The experiments were well designed and completed. Here are some issues to be addressed

1. It is interesting that TIGIT specifically blockade could break immune tolerance in HBs-tg mice. Is there any clinical data showed that TIGIT molecule is associated with the development of hepatitis or HCC? For example, TIGIT+CD8+ T cells increase as HCC progresses? Any data including TCGA show the correlation between increased TIGIT and worse prognosis?
2. In Fig.4, as PD-1 inhibitory receptor increased similarly as TIGIT with age in HBs-tg mice. Could PD-1 or PD-L1 blockade also be enough to break T cells tolerance and induce hepatitis or HCC?
3. TIGIT play an inhibitory role in NK cells. In Supplemental Fig.3, Could the depletion of NK cells affect the hepatitis development induced by TIGIT blockade?
4. The cellular and molecular mechanisms of CD8+ T cell mediated hepatitis and HCC development need to be explained or discussed more. Such as, Is IFNg from T cells involved in this process?

Reviewer #2:
Remarks to the Author:
Tian et al. explored the possible role of CD8+ T cells in adaptive immunity-mediated tumor progression in a HBsAg transgenic mouse model. The data showed that breaking CD8+ T cell tolerance by TIGIT blockade resulted in chronic hepatitis and fibrosis. Meanwhile, HBsAg vaccination combing TIGIT blockade triggered HCC development. Generally, the study provided interesting findings connecting adaptive immunotolerance and HCC. But for publication purpose, the paper needs to clarify the following points.
Major points:
1. In figure 1c, infiltration of leukocytes in the liver tissue was shown. But whether these cells are CD8+ T cells or not was not examined. In figure 1e, the results of TUNEL staining was not convincing. Representative figures and statistical analysis are to be supplemented.
2. In figure 2d, the structure of tumor in a-TIGIT+vaccine group was hardly to tell from other benign hyperplastic nodules. The authors need to provide more evidence to confirm the cancerous features of this entity (such as AFP staining, genetic analysis etc.) This also applies to Fig 3C.
3. In figure 6g-j, the data showed the responses of CD8+ T cells to PMA/Ionomycin stimulation. Did the study evaluate how these cells respond to HBV antigen pool?
4. In figure 8j, the quality of figure showing HCC needs to be improved.
5. The authors showed histological alterations regarding hepatitis and fibrosis after TIGIT blockade in HBs-tg mice. What are the changes of liver after intervention with TIGIT blockade combining HBsAg vaccination, especially in the phase of vaccination before tumor appearance? This also applies to CD8+ T cell depletion experiments, where the changes of liver in the aspects of inflammation and fibrogenic development during depletion phase was important for understanding the end point findings.
6. In-depth exploration on the underlying mechanisms responsible for CD8 competence and hepatitis/HCC development in this HBsAg transgenic mouse model is to be supplemented.

Minor points:
1. In page6, paragraph 2, line 9:
“In the boundary zone between tumor and normal tissue,”----to be accurate, here “normal” may be replaced by “non-tumor” .

2. In page6, paragraph 3, line 3:
“and the hepatocytes in tumor regions”----here “hepatocytes” may be replaced by “cells”

3. In page 12, paragraph 5:
“liver sections were fixed in 10% acidic buffered formalin”----“acidic” or “neutral”?

Reviewer #3:
Remarks to the Author:
In the present manuscript, Zong et al. describe that TIGIT is highly expressed on intrahepatic CD8+ T cells in HBsAg-tg mice. TIGIT blockade or deficiency led to chronic hepatitis and fibrosis in these mice probably by a break of adaptive immune tolerance. Additional HBsAg vaccination led to HCC development in a CD8+ T-cell-dependent manner in TIGIT-deficient HBsAg-tg mice. Thus, the authors not only made the observation that abrogating a single inhibitory pathway may lead to an accelerated (HBsAg-tg mice develop similar symptoms with age) break of tolerance in HBsAg-tg mice but have also developed a mouse model of HBV-related HCC. The study is experimentally sound and the paper is well written. However, the conclusions made are broadly generalized and therefore not always fully supported by the data shown in the manuscript.
I have the following comments:
Major points:
1.) The heading “Breakdown of adaptive immune tolerance induces hepatocellular carcinoma” is overgeneralized since the analyzed mouse model is based on tolerance induction. “Breakdown of adaptive immune tolerance induces hepatocellular carcinoma in HBsAg-tg mice” would be more appropriate.
2.) Is HCC development only observed in HBsAg-tg mice following vaccination and TIGIT blockade or also in association with blockade of other inhibitory, e.g. PD-1, CTLA-4, etc.?
3.) Is there evidence for compensatory responses in HBsAg-tg Tigit-/- mice compared to HBsAg-tg mice as it has been checked for mAb-mediated TIGIT blockade? This may be a reason for the discrepancy between TIGIT blockade and deficiency (Fig.5/6 and Fig.7) with respect to T cell frequencies in liver and spleen.
4.) Data in Fig.1E are depicted in a representative manner. Please quantitate the data shown in Fig.1E.
5.) Fig.2 and Fig.3: The course of HCC development in HBsAg-tg mice with abrogated TIGIT pathway and HBsAg vaccination is presented in a limited manner. Please provide histological analyses performed at later time-points, ALT levels (time-course), body weight (time-course) and survival.
6.) Fig.6a: In tissues, CD69 expression also marks tissue-residency. Please support the conclusion of increased intrahepatic CD8+ T-cell activation after TIGIT blockade by applying other markers (e.g. Ki67, etc.).
7.) Fig.6: With respect to an overall increase in T cells in the liver, please provide absolute cell numbers for CD69+, CD44hiCD62Llow, CD127+CD62L+ CD8+ T cells.
Minor points:
1.) Fig.5C: Please do not pre-gate on CD8+. Showing CD8 vs HBsAg-tetramer also on CD8- cells will help to evaluate the quality of the HBsAg-tetramer staining.
2.) Fig.6G/6I: Please show negative controls (w/o PMA/Ionomycin stimulation) and do not pre-gate on CD8+.
3.) Page 7, line 25: Please be precise in applying the concept of exhaustion and tolerance. These are two different immunological situations.,

Dear editor:

We submitted our manuscript entitled "Breakdown of adaptive immunotolerance induces hepatocellular carcinoma" (ref. num. NCOMMS-18-08367) to Nature Communications four months ago. Although it was not accepted in its former form, you kindly give us a chance to revise our manuscript. We re-revised the manuscript with some new results to answer the questions or address the concerns raised by the Reviewers.

Response to Comments from the Editors. In your decision letter on May 15, 2018, you pointed out that several aspects of the paper needed to be resolved. In particular, **1)** to provide further and extensive experimental data to support the clinical relevance of our findings by showing that the TIGIT molecule is expressed and associated with the development of hepatitis or HCC (reviewer#1/point1). **2)** to provide further data dissecting the molecular mechanism through which CD8+ T cell mediates hepatitis and HCC development (Reviewer#1/point4 and Reviewer#2/point6). **3)** Further explanations and/or further data supporting the general validity of the conclusions of this manuscript to HBV-related HCC (Reviewer#3/point1).

We carefully addressed the above points in the new version. First, we provide clinical data of HCC from the TCGA database to show that *TIGIT* gene expression of intratumoral tissue was higher than paratumor tissue (answer to Reviewer#1) and *TIGIT* gene expression is positively correlated with *PDCD1* (PD-1) gene in tumor tissue (**Fig. 9a)**. Besides, we compared TIGIT expression between chronic HBV (CHB) patients and healthy controls, and found higher TIGIT expression on peripheral blood $CD8^+$ T cells in CHB patients than in healthy controls (**Fig. 9b**). These results suggest a clinical relevance of our findings obtained from mouse models.

To gain a better understanding of the molecular mechanism through which $CD8^+$ T cell mediates hepatitis and HCC development, experiments were conducted to evaluate human and mouse $CD8^+$ T cell responses to HBV peptides after TIGIT blockade *in vitro*. We found that TIGIT blockade in vitro enhanced cytokine production and cytotoxicity of $CD8^+$ T cells from CHB patients and HBs-tg mice upon HBV peptide stimulation (**Fig. 9c, d and Supplementary Figure 4**). Moreover, we did observe $CD8^+$ T cell infiltration in the liver of TIGIT-blocked HBs-tg mice (**Supplementary Figure 2d).** We speculate that $CD8^+$ T cells might promote hepatitis and HCC initiation through producing inflammatoty cytokines and direct cytotoxicity to hepatocytes, both of which can induce hepatocyte death and amplify inflammatory responses, while TIGIT expression on $CD8^+$ T cells could inhibit liver inflammation and thereby delay HCC initiation. In the revised manuscript, we discussed the potential mechamisms of TIGIT and $CD8^+$ T cells in hepatitis and HCC development in the discussion (**paragragh 3 and paragragh 4**).

Finally, we accept the suggestion of Reviewer#3/point1 and change our title into "Breakdown of adaptive immunotolerance induces hepatocellular carcinoma in HBsAg-tg mice". These changes were highlighted in the revised version of manuscript.

Response to the Reviewers' comments point-by-point
Reviewer #1

1. It is interesting that TIGIT specifically blockade could break immune tolerance in HBs-tg mice. Is there any clinical data showed that TIGIT molecule is associated with the development of hepatitis or HCC? For example, TIGIT+CD8+ T cells increase as HCC progresses? Any data including TCGA show the correlation between increased TIGIT and worse prognosis?

Response:

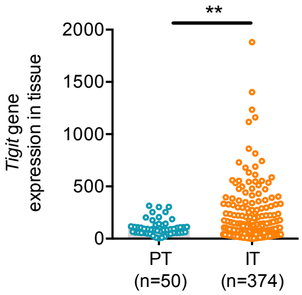

***TIGIT* gene expression of intratumoral tissue was higher than paratumor tissue.**

Legend: *TIGIT* gene expression levels in IT (N=374) and PT (N=50) of HCC patients from the TCGA database (Mann-Whitney non-parametric statistical test Unpaired t test). Each dot represents an individual patient. The results are expressed as the mean ± SEM.

We sincerely thank the reviewer for the valuable comments. To investigate the clinical relevance of our findings, we analyzed clinical data of HCC from the TCGA database and found that *TIGIT* gene expression in intratumoral tissue was higher than in paratumor tissue (**Figure shown above**). Moreover, *TIGIT* expression is positively correlated with *PDCD1* expression in HCC tumor tissues (**Fig. 9a)**. Besides, we found that surface expression of TIGIT on CD8$^+$ T cells in chronic HBV (CHB) patients, was higher than in healthy controls (**Fig. 9b**). Peripheral blood mononuclear cells (PBMCs) from CHB patients were isolated , incubated with anti-TIGIT mAb or control mouse IgG for 45 min, and then stimulated with HBsAg peptide for 10 days *in vitro* . Increased expression of IFN-γ , CD107a, TNF-α and Ki67 in CD8$^+$ T cells were observed in TIGIT-blocked group (**Fig. 9c, d)**. These results are in consistent with the findings obtained from our mouse models (**Supplementary Figure 4**).

Regarding the relationship between TIGIT expression and HCC, it is possible that TIGIT may play dual roles in different stages of tumor development. In this manuscript, we focus on the roles of TIGIT in the initiation of HBV-related HCC. Our results suggest that, before tumor appearance, TIGIT induces CD8$^+$ T cell dysfunction and maintains hepatic immune tolerance in HBV transgenic mice, thereby inhibiting immune-mediated injury and delaying tumor initiation. However, our recent published study reveal that increased TIGIT expression could promote tumor progression by dampening effector lymphocyte-mediated anti-tumor immunity in tumor-bearing mice (Zhang Q et al, Nat Immunol 2018). In line with this, the use of checkpoint inhibitors have shown clinical benefits in patients with advanced cancer. Therefore, TIGIT expression on CD8$^+$ T cells before and after HCC occurrence may have different effects on tumor development. In the new version of manuscript, we discussed this point in the discussion (**paragragh 3**).

2. In Fig.4, as PD-1 inhibitory receptor increased similarly as TIGIT with age in HBs-tg mice. Could PD-1 or PD-L1 blockade also be enough to break T cells tolerance and induce hepatitis or HCC?

Response:

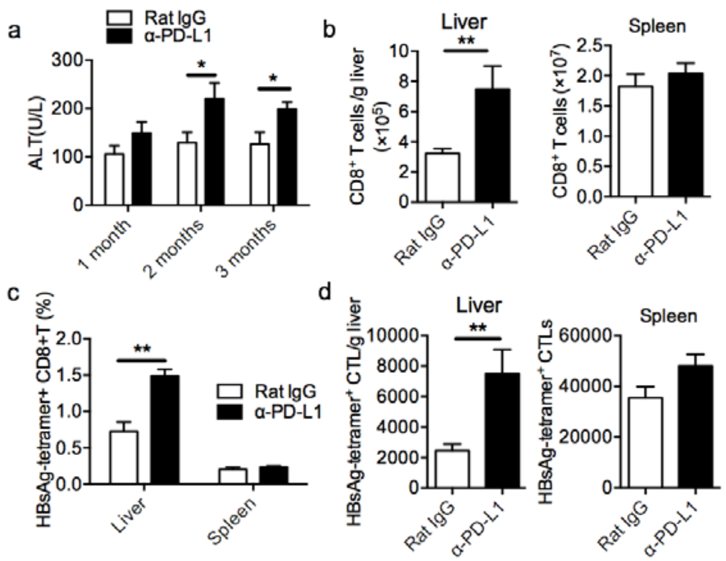

PD-L1 blockade also mediates the liver injury and increases HBsAg-specific CTLs in liver.

Legend: (**a-d**) Two groups of HBs-tg mice were intraperitoneally injected with 200 μg anti-PD-L1(clone: 10F.9G2) and 200 μg control rat IgG weekly respectively. (**a**) Serum ALT levels at different time points after start of antibody treatment in HBs-tg. (**b**) The absolute number of hepatic and splenic CD8+ T cells in HBs-tg mice treated with α-PD-L1 mAb or rat IgG for 3 months is shown. (c)The statistical percentages of HBsAg-specific CTLs in the liver and spleen of two groups of HBs-tg mice. (**d**) The absolute number of hepatic and splenic HBsAg-specific CTLs in HBs-tg mice treated with α-PD-L1 mAb or rat IgG for 3 months is shown. 4 to 6 mice per group. Statistically significant differences between the groups are presented as the mean ± SEM.: *P＜0.05; **P＜0.01.

We are also interested in this question. And we blocked PD-1-PD-L1 pathway in HBs-tg mice for 3 months. As shown in the figure above, anti-PD-L1 mAb injection also increased the ALT levels in HBs-tg mice (**upper Fig. a**). We examined the frequency and absolute number of hepatic $CD8^+$ T cells and HBsAg-speicifc CTLs **(upper Fig.b-d**), and found that they also significantly increased. However, due to the limited time frame for revising this manuscript, we didn't go on to see if these mice get HCC toward HBsAg vaccination. Considering that hepatitis and HBsAg-specific CTLs are the essential requirements for inducing HCC in our mouse model, it is likely that PD-1 pathway blockade can also induce HCC in HBs-tg mice.

3. TIGIT play an inhibitory role in NK cells. In Supplemental Fig.3, Could the depletion of NK cells affect the hepatitis development induced by TIGIT blockade?

Response:

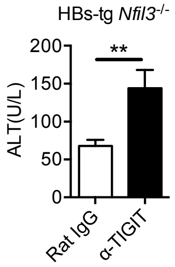

NK cell-deficient HBs-tg mice exhibit increased ALT levels after TIGIT blockade.

Legend: Breeding HBs-tg *Nfil3*$^{-/-}$ mice were intraperitoneally injected with 200 μg α-TIGIT mAb (clone 13G6) or control rat IgG weekly for 1month. Serum ALT levels 1 months after start of antibody treatment in HBs-tg *Nfil3*$^{-/-}$

mice. 3 or 5 mice per group. Statistically significant differences between the groups are presented as the mean ± SEM.: **P＜0.01.

Nfil3$^{-/-}$ mice are deficient in NK cells, our lab has generated HBs-tg *Nfil3*$^{-/-}$ mice by crossing HBs-tg mice and *Nfil3*$^{-/-}$ mice. We injected anti-TIGIT mAb or rat IgG to HBs-tg *Nfil3*$^{-/-}$ mice weekly. After 1 month, the ALT levels were significantly higher than those in rat IgG-treated mice (**Figure above**). Limited by the time, we didn't examine ALT levels in HBs-tg *Nfil3*$^{-/-}$ mice after TIGIT blockade at a longer time. Nevertheless, these data suggest that HBs-tg mice still develop chronic hepatitis in the absence of NK cells, and NK cells may not affect the role of HBsAg-specific CTLs in TIGIT blocking-induced hepatitis.

4. The cellular and molecular mechanisms of CD8+ T cell mediated hepatitis and HCC development need to be explained or discussed more. Such as, Is IFNg from T cells involved in this process?

Response:

We observed that TIGIT blockade enhanced cytokine production (IFN-γ, TNF-a) and cytotoxicity (CD107a) of CD8$^+$ T cells from CHB patients and HBs-tg mice upon HBV peptide stimulation (**Fig. 9c, d and Supplementary Figure 4**). We speculate that CD8$^+$ T cells may mediate hepatitis and HCC development mainly by two mechanisms. First, CD8$^+$ T cell secreted multiple inflammatory cytokines such as IFN-γ, which can induce hepatocyte apoptosis (*J Hepatol* 2012, 430-441). Wang et al. have reported that CD8$^+$ T cell-derived IFN-γ induces fibrosis-promoting cytokines/chemokines in the HBV-related HCC pathogenesis (*J Immunol*.2010, 7654-7662). Second, HBsAg-specific CTLs could kill HBsAg-expressing hepatocytes directly via FASL (*Gastroenterology* .2014, 765-783 e764). Persistent hepatocyte apoptosis may cause the release of lots of multiple apoptosis-associated proteins. Thus, the inflammatory responses could be further amplified and many other kind of inflammatory cell types were recruited to the liver (*J Exp Med.* 2001, 1755-1766). We have shown that many leukocytes infiltrated to liver after TIGIT blockade. However, only a proportion of the infiltrated cells were CD8$^+$ T cells, suggesting infiltration of many other cell types (**Supplementary Figure 2d)**. The chronic inflammation might be a crucial driving mechanism for the development of HCC. Furthermore, massive hepatocellular death is usually accompanied by continuous cell turnover, which also accelerates the process of carcinogenesis. For these complicated events, HCC occurred finally. In the revised manuscript, we discussed this point in the discussion (**paragragh 4**).

Reviewer #2

Major points:

1 In figure 1c, infiltration of leukocytes in the liver tissue was shown. But whether these cells are CD8+ T cells or not was not examined. In figure 1e, the results of TUNEL staining was not convincing. Representative figures and statistical analysis are to be supplemented.

Response:

Thanks for your advice. In the revised manuscript, we performed immunofluorescence staining and showed that the infiltrated cells contained CD8 positive cells (**Supplementary Fig. 2d**). There were also CD8 negative cells among the inflilitrated cells, and they may be macrophages and neutrophils recruited to liver. For TUNEL staining, we repeated the experiment and provided representative figures with higher resolution and quality and added statistical analysis in **Fig1e, f**.

2. In figure 2d, the structure of tumor in a-TIGIT+vaccine group was hardly to tell from other benign hyperplastic nodules. The authors need to provide more evidence to confirm the cancerous features of this entity (such as AFP staining, genetic analysis etc.) This also applies to Fig 3C.

Response:

According to the reviewer's suggestion, we performed AFP staining of liver sections from vaccinated HBs-tg mice after TIGIT blockade or rat IgG treatment. Among the tumor region, there are AFP positive cells (**Fig.2e**). Quantative PCR analysis also revealed that *Afp* mRNA level was higher in a-TIGIT+vaccine group than rat IgG+vaccine group (**Fig.2f**). Similar results were obtained when comparing HBs-tg *Tigit* $^{-/-}$ group with HBs-tg (**Fig**.**3d, e)**.

3. In figure 6g-j, the data showed the responses of CD8+ T cells to PMA/Ionomycin stimulation. Did the study evaluate how these cells respond to HBV antigen pool?

Response:

We evaluated $CD8^+$ T cell responses to HBV antigen and added the results in the revised version (**Supplementary Fig. 4a, b**). Isolated lymphocytes from HBs-tg mice were blocked by anti-TIGIT mAb *in vitro*. Then they were expanded for 4 days by stimulation with HBsAg peptide. Increased expression of IFN-γ, CD107a, TNF-α and IL-2 were observed in TIGIT-blocked samples. The frequency of $Ki67^+CD8^+$ T cells also increased after TIGIT blockade (**Supplementary Fig. 4a,b)**. This *in vitro* experiment shows TIGIT blockade restores $CD8^+$ T cell function directly.

4. In figure 8j, the quality of figure showing HCC needs to be improved.

Response:

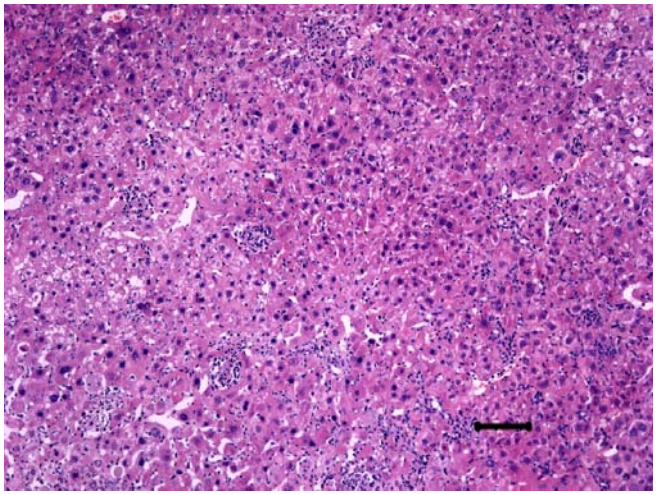

HCC picture of **Fig.8j** (Scale bar, 100 μm.)

In the revised version, we added H&E staining pictures in a larger scale (**Fig.8j**). From this picture, typical trabecular HCC features, and destructive liver architecture can be seen. Moreover, there are numerous infiltrated cells.

5. The authors showed histological alterations regarding hepatitis and fibrosis after TIGIT blockade in HBs-tg mice. What are the changes of liver after intervention with TIGIT blockade combining HBsAg vaccination, especially in the phase of vaccination before tumor appearance? This also applies to $CD8^+$ T cell depletion experiments, where the changes of liver in the aspects of inflammation and fibrogenic development during depletion phase was important for understanding the end point findings.

Response:

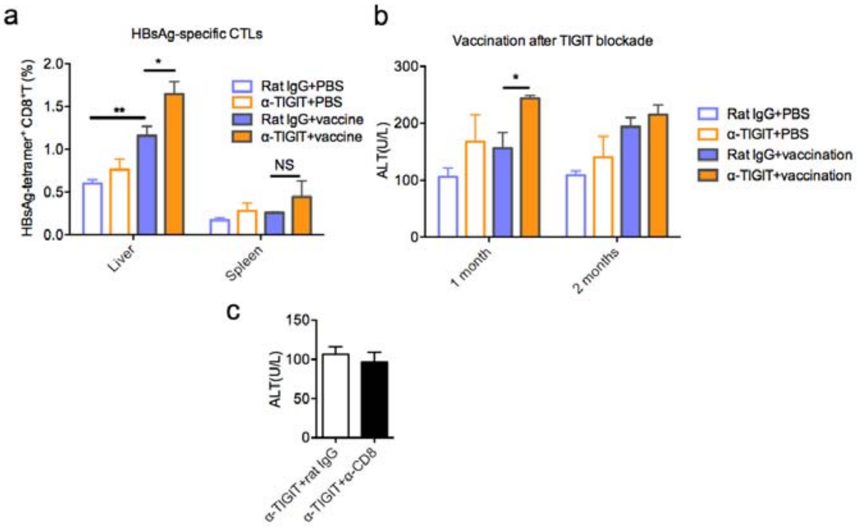

Vaccination to TIGIT-blockade mice increases HBs-specific CTLs in liver.

Legend: (**a,b**) HBs-tg mice were intraperitoneally injected with 200 μg α-TIGIT mAb or rat IgG weekly for 3 months, then followed by intramuscular injection with 1 μg HBsAg vaccine or 50 μl PBS biweekly. (**a**) The statistical percentages HBsAg-specific CTLs in the liver and spleen of rat IgG or α-TIGIT-treated HBs-tg mice after 2-month vaccination. **(b)** Serum ALT levels at different time points after start of vaccination in HBs-tg mice. 3 to 5 mice per group. Statistically significant differences between the groups are presented as the mean ± SEM.: *P＜0.05; **P＜0.01. (**c**) HBs-tg mice were intraperitoneally injected with 200μg α-TIGIT mAb weekly for 3 months, followed by weekly intraperitoneal injection with 100μg CD8$^+$ T cell depleting antibody (α-CD8) or rat IgG until harvest. At the 13th week, mice were intramuscularly injected with 1 μg HBsAg vaccine biweekly. All mice were harvested 2 months after the final vaccination. Serum ALT levels at harvest.

We harvested mice after 2-month vaccination, and found higher levels of HBsAg-specific CTLs in the liver of α-TIGIT+vaccine group than rat IgG+vaccine group **(upper fig. a**). One month after start of vaccination (vaccinated twice), ALT levels significantly increased in the α-TIGIT+vaccine group **(upper fig. b**). Two months after start of vaccination (vaccinated fourth), a slight upward trend of ALT levels in the α-TIGIT+vaccine group compared to the IgG-treated group was observed, but there were no statistically significant differences. The narrowing gap of ALT levels at the second month might be due to the elevated ALT levels in the rat IgG+vaccine group, as increasing times of vaccination might stimulate CD8$^+$ T cell responses in this group. The HBs-tg mice were not absolute tolerant mice. They have been reported to spontaneously develop HCC from 14- to 16-months of age, with a disease prevalence of ~40%. As shown in the figure above **(upper fig. a**), rat IgG+vaccine group also harbors higher HBsAg-specific CTLs compared to rat IgG+PBS group. As for CD8$^+$ T cell depletion experiments, we are sorry that we had not analyzed inflammation and fibrogenic development at an earlier stage during depletion phase. If we start this experiment again, it would take at least five months to finish it. Considering that we found there was no hepatitis and HCC development two months after the final vaccination in CD8 T cell-depleted mice (**Fig.8j** and **upper fig. d**), it is possible that these mice would have no hepatitis or fibrogenic development at an earlier stage.

6. In-depth exploration on the underlying mechanisms responsible for CD8 competence and hepatitis/HCC development in this HBsAg transgenic mouse model is to be supplemented.

Response:

To gain a better understanding of the molecular mechanism through which $CD8^+$ T cell mediates hepatitis and HCC development, experiments were conducted to evaluate human and mouse $CD8^+$ cell responses to HBV peptides after TIGIT blockade *in vitro*. We found that TIGIT blockade enhanced cytokine production and cytotoxicity of $CD8^+$ T cells from CHB patients and HBs-tg mice upon HBV peptide stimulation (**Fig. 9c, d** and **Supplementary Figure 4**). We speculate that $CD8^+$ T cells might promote hepatitis and HCC initiation through producing inflammatoty cytokines and direct cytotoxicity to hepatocytes (*J Hepatol* 2012, 430-441; *Gastroenterology*.2014, 765-783 e764), both of which can induce hepatocyte death and amplify inflammatory responses (*J Exp Med.* 2001, 1755-1766). There is accumulating evidence showing that chronic inflammation is crucial to tumor development *(Gastroenterology*.2014, 765-783 e764). Meanwhile, uncontrolled hepatocellular death is usually accompanied by continuous cell turnover, which also accelerates the process of carcinogenesis. Since the effector molecules of $CD8^+$ T cells include multiple cytokines and cytotoxic molecules, which might have synergistic effects on promoting hepatitis and HCC initiation, it would be hard for us to find out which effector molecules are critical in this process. In the revised version of manuscript, we discussed the potential mechanisms responsible for $CD8^+$ T cells in hepatitis and HCC in the discussion (**paragragh 4**).

Minor points:

1. In page6, paragraph 2, line 9:

"In the boundary zone between tumor and normal tissue,"----to be accurate, here "normal" may be replaced by "non-tumor" .

Response:

As advised by the reviewer, we have modified it in the revised version.

2. In page6, paragraph 3, line 3:

"and the hepatocytes in tumor regions"----here "hepatocytes" may be replaced by "cells"

Response:

As advised by the reviewer, we have modified it in the revised version.

3. In page 12, paragraph 5:

"liver sections were fixed in 10% acidic buffered formalin"----"acidic" or "neutral"?

Response:

It was "neutral". We have modified it in the revised version.

Reviewer #3

Major points:

1) The heading "Breakdown of adaptive immune tolerance induces hepatocellular carcinoma" is overgeneralized since the analyzed mouse model is based on tolerance induction. "Breakdown of adaptive immune tolerance induces hepatocellular carcinoma in HBsAg-tg mice" would be more appropriate.

Response:

Thanks for the suggestion. We have changed the title according to the suggestion.

2.) Is HCC development only observed in HBsAg-tg mice following vaccination and TIGIT blockade or also in association with blockade of other inhibitory, e.g. PD-1, CTLA-4, etc.?

Response:

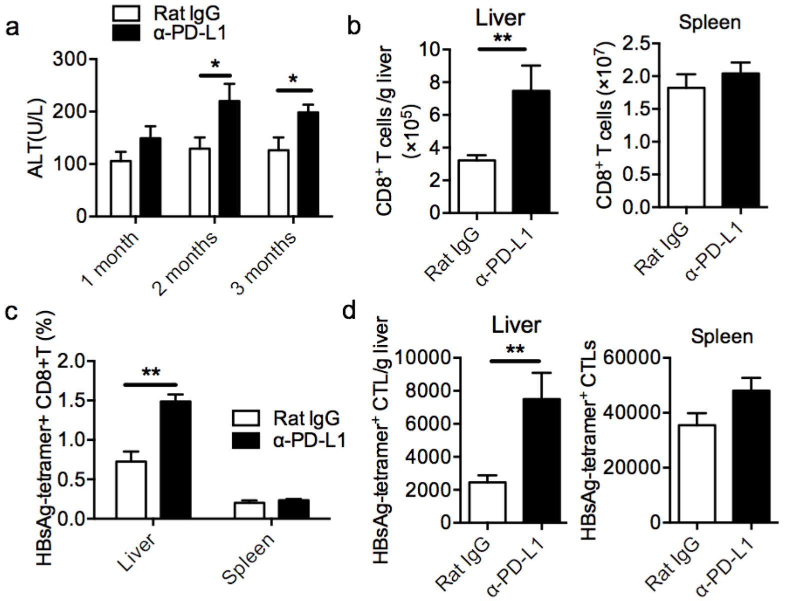

PD-L1 blockade also mediates the liver injury and increases HBsAg-specific CTLs in liver.

Legend: (**a-d**) Two groups of HBs-tg mice were intraperitoneally injected with 200 µg anti-PD-L1(clone: 10F.9G2) and 200 µg control rat IgG weekly respectively. (**a**) Serum ALT levels at different time points after start of antibody treatment in HBs-tg. (**b**) The absolute number of hepatic and splenic CD8+ T cells in HBs-tg mice treated with α-PD-L1 mAb or rat IgG for 3 months is shown. (**c**)The statistical percentages of HBsAg-specific CTLs in the liver and spleen of two groups of HBs-tg mice. (**d**) The absolute number of hepatic and splenic HBsAg-specific CTLs in HBs-tg mice treated with α-PD-L1 mAb or rat IgG for 3 months is shown. 4 to 6 mice per group. Statistically significant differences between the groups are presented as the mean ± SEM.: *P＜0.05; **P＜0.01.

We are also interested in this question. And we blocked PD-1-PD-L1 pathway in HBs-tg mice for 3 months. As shown in the figure above, anti-PD-L1 mAb injection also increased the ALT levels in HBs-tg mice (**upper Fig.a**). We examined the frequency and absolute number of hepatic $CD8^+$ T cells and HBsAg-speicifc CTLs (**upper Fig.b-d**), and found that they also significantly increased. However, due to the limited time frame for revising this manuscript, we didn't go on to see if these mice get HCC toward HBsAg vaccination. Considering that hepatitis and HBsAg-specific CTLs are the essential requirements for inducing HCC in our mouse model, it is likely that PD-1 pathway blockade can also induce HCC in HBs-tg mice.

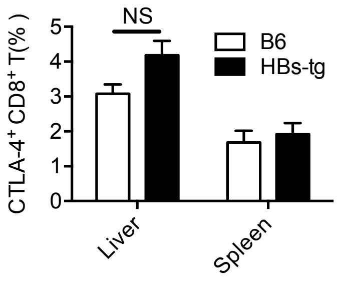

$CD8^+$ T cells in HBs-tg mice express low levels of CTLA-4

Legend: CTLA-4 expression on $CD8^+$ T cells in the liver and spleen of 10-month-old WT and HBs-tg mice. 5 mice per group. Data are presented as the mean ± SEM.

As for CTLA-4, we examined 10-month-old WT and HBs-tg mice, and found that CTLA-4 expression on liver $CD8^+$ T cells in HBs-tg mice displayed no differences compared to that in WT mice. Moreover, only about 4% of liver $CD8^+$ T cells expressed CTLA4 (**Figure above**), a much lower level than PD-1(~15%) and TIGIT (~25%) (**Fig. 4c, d**). Based on these results, we considered that TIGIT and PD-1 might be more important than CTLA-4 in maintaining the tolerance of HBs-tg mice.

3.) Is there evidence for compensatory responses in HBsAg-tg Tigit-/- mice compared to HBsAg-tg mice as it has been checked for mAb-mediated TIGIT blockade? This may be a reason for the discrepancy between TIGIT blockade and deficiency (Fig.5/6 and Fig.7) with respect to T cell frequencies in liver and spleen.

Response:

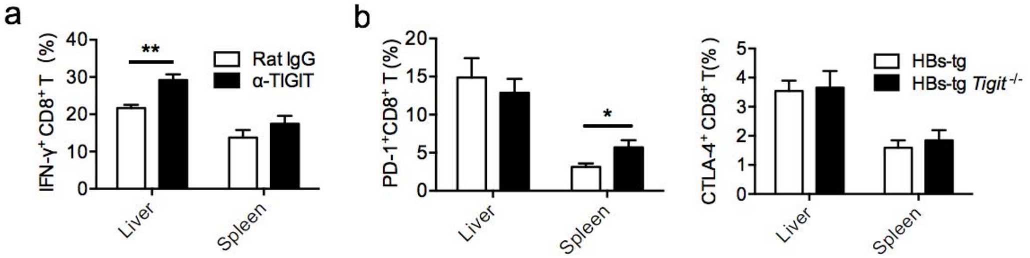

Unaltered expression of PD-1 and CTLA-4 on hepatic $CD8^+$ T cells in *$Tigit^{-/-}$* HBs-tg mice

Legend: (**a**) HBs-tg mice were treated with rat IgG or α-TIGIT mAb for 3 months. Expression of intracellular IFN-γ by hepatic and splenic $CD8^+$ T cells of mice after *ex vivo* stimulation with PMA and ionomycin. (**b**) CTLA-4 expression on $CD8^+$ T cells in the liver and spleen of 10-month-old HBs-tg mice and HBs-tg *$tigit^{-/-}$* mice. 5 or 6 mice per group. Statistically significant differences between the groups are presented as the mean ± SEM: $^{**}P < 0.01$.

In TIGIT blockade experiment, the percentages of IFN-γ^+CD8^+ T cells among $CD8^+$ T cells rose from 21% to 30% (**upper Fig.a**) (This data is from previous version, the revised version was counted on IFN-γ^+CD8^+ cells among total T cells, **Fig 6k, l**); but in knockout mice, IFN-γ production rose from 28% to 57% (**Fig.7c,d**). We think it may be a reason that HBs-tg *$Tigit^{-/-}$* mice develop hepatitis in spite of no higher proportion of HBsAg-specific CTLs and $CD8^+$ T cells detected in liver. To investigate whether other surface receptors compensatory for *Tigit* deficiency, we also examined the inhibitory receptor PD-1 and CTLA4 before. But we found that PD-1 and CTLA-4 did not increase its expression on hepatic $CD8^+$ T cells in *$Tigit^{-/-}$* HBs-tg mice (**upper Fig.b**).

4.) Data in Fig.1E are depicted in a representative manner. Please quantitate the data shown in Fig.1E.

Response:

According to the suggestion, we repeated the experiment and the statistical analysis is shown in **Fig1e, f**.

5.) Fig.2 and Fig.3: The course of HCC development in HBsAg-tg mice with abrogated TIGIT pathway and HBsAg vaccination is presented in a limited manner. Please provide 1histological analyses performed at later time-points, 2ALT levels (time-course), 3body weight (time-course) and 4survival.

Response:

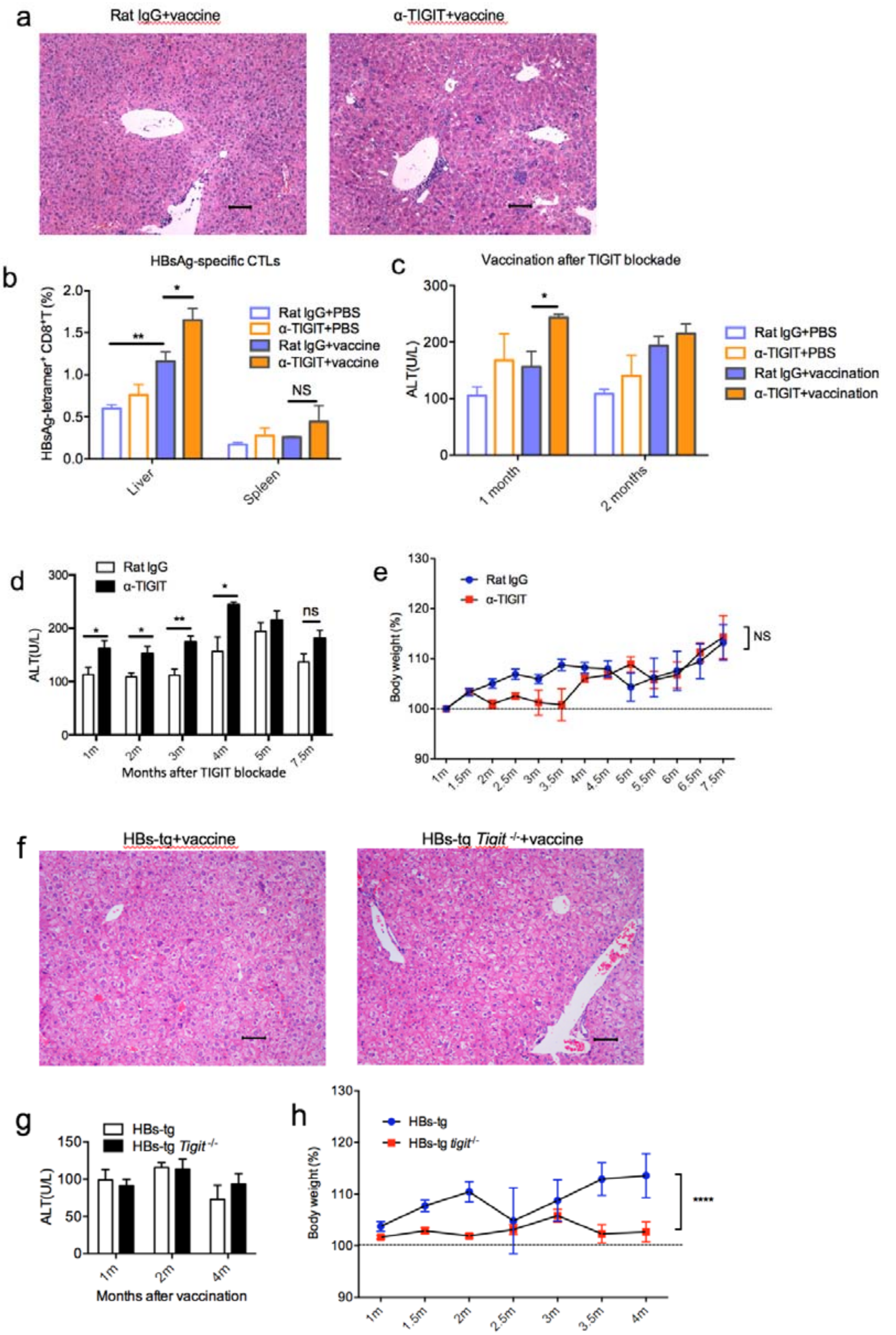

Legend: (**a-c**) HBs-tg mice were intraperitoneally injected with 200 µg α-TIGIT mAb or rat IgG weekly for 3 months, then followed by intramuscular injection with 1 µg HBsAg vaccine or 50 µl PBS biweekly. (**a**) The statistical percentages HBsAg-specific CTLs in the liver and spleen of rat IgG or α-TIGIT-treated HBs-tg mice after 2-month vaccination. **(b)** Serum ALT levels at different time points after start of vaccination in HBs-tg mice. (**c**) Representative H&E of liver tissue sections in HBs-tg mice after 2-month vaccination. Scale bar, 100 µm. 3 to

5 mice per group. Statistically significant differences between the groups are presented as the mean ± SEM.: *P< 0.05; **P<0.01. (**d-e**) HBs-tg mice were intraperitoneally injected with 200μg α-TIGIT mAb or rat IgG weekly for 3 months. At the 13th week, mice were intramuscularly injected with 1 μg HBsAg vaccine biweekly. All mice were harvested 2 months after the final vaccination. ALT level(**d**) and body weight change(**e**) at different time points after start of antibody treatment. Statistically significant differences (**d,** unpaired T test**; e,** two-way ANOVA test) between the groups are presented as the mean ± SEM.: *P<0.05; **P<0.01.
(**f**) HBs-tg mice and HBs-tg *Tigit*-/- mice were intramuscular injected with 1 μg HBsAg vaccine or 50 μl PBS biweekly with four consecutive repetitions. Mice were harvested after the final vaccination. H&E of liver tissue sections the mice after the final vaccination. Scale bar, 100 μm. (**g,h**) 6-month-old HBs-tg *Tigit*$^{-/-}$ mice and control HBs-tg mice were intramuscularly injected with 1 μg HBsAg vaccine or 50 μl PBS biweekly with consecutive repetitions. All mice were harvested 2 months after the final vaccination. ALT level(**g**) and body weight change(**h**) at different time points after start of vaccination. Statistically significant differences (**g,** unpaired T test**; h,** two-way ANOVA test) between the groups are presented as the mean ± SEM.: *P **<** 0.05; **P **<** 0.01.

1) We harvested mice after the final vaccination before HCC occurrence, and the H-E staining showed infiltrated lymphocytes in the liver **(upper fig. a**). We also found higher levels of HBsAg-specific CTLs in the liver of α-TIGIT+vaccine group than rat IgG+vaccine group **(upper fig. b**). One month after start of vaccination (vaccinated twice), ALT levels significantly increased in the α-TIGIT+vaccine group (**upper fig. c**). Two months after start of vaccination (vaccinated fourth), a slight upward trend of ALT levels in the α-TIGIT+vaccine group compared to the IgG-treated group was observed, but there was no statistically significant differences. The narrowing gap of ALT levels at the second month might be due to the elevated ALT levels in the rat IgG+vaccine group, as increasing times of vaccination might stimulate CD8^{+} T cell responses in this group **(upper fig. b**). The HBs-tg mice were not absolute tolerant mice. They have been reported to spontaneously develop HCC from 14- to 16-months of age, with a disease prevalence of ~40%. For knockout mouse model, infiltrated lymphocytes can also be seen in the liver after vaccination **(upper fig. f**). However, we are sorry that we had not previously analyzed these mice at a later stage during HCC progression, as we focused on the events before HCC initiation.
2) The ALT levels of HBs-tg mice starting from the first time of anti-TIGIT mAb injection till HCC occurrence is shown in **(upper fig. d**). ALT levels was higher than rat IgG+vaccine group during the TGIT blockade period till the second vaccination. However, ALT levels decreased to the levels of control group when HCC happened, which is similar to chronic HBV-induced HCC in other mouse model (*J Exp Med.* 1998, 341-350). HBs- tg *Tigit*$^{-/-}$ mice didn't harbor higher ALT levels than HBs-tg after vaccination **(upper fig. g**), as explained in the above paragraph in **1)**.
3) The body weight change of HBs-tg mice starting from the first time of anti-TIGIT mAb injection till HCC occurrence is shown in **(upper fig. d**). The body weight was lower in α-TIGIT+vaccine group than in rat IgG+vaccine group during the TGIT blockade period till the second vaccination. At later time, there was no differences between these groups, and we think that the liver weight increase in the TIGIT blockade group might affect the tendency of whole body weight changing. We found that the liver sometimes weighed 3-4 g when HCC happened. For the HBs- tg *Tigit*$^{-/-}$ mice, the body weight changing was constantly lower than HBs-tg mice **(upper fig. h**). As shown in **(Fig. 3a**), the liver size was small and didn't affect the tendency of whole body weight changing.
4) We are sorry that we don't have survival results due to the limited time frame for revising this manuscript. In both the TIGIT blockade and knocknout mouse models, we observed the mice at about 9.5-10-month old when HCC happened. During this time period, there were no mice died.

But it is likely that there will be significant changes in survival rates between the two groups, if we prolong the observation time.

6.) Fig.6a: In tissues, CD69 expression also marks tissue-residency. Please support the conclusion of increased intrahepatic CD8+ T-cell activation after TIGIT blockade by applying other markers (e.g. Ki67, etc.).
Response:

Thanks for your suggestion. CD69 indeed marks tissue-residency and lymphocyte activation (*Nature Reviews Immunology* 2014, 619-629). To confirm the activation state of $CD8^+$ T cells after TIGIT blockade, we also examined Ki67 expression as advised by the reviewer. We found the frequency and absolute number of $Ki67^+CD8^+$ T cells (**Fig. 6e, j** and **Supplementary Fig. 3d**) increased dramatically in the liver after TIGIT blockade. Moreover, expression of CD25, another activation marker, was also up-regulated, although its overall expression is relatively low **(Fig. 6b, g**). In addition, hepatic $CD8^+$ T cells from the TIGIT-blockade group also exhibited high expression of CD44, which can be used as a memory or activation marker (**Fig. 6c, h**). These results collectively suggest that hepatic $CD8^+$ T cells are activated after TIGIT blockade.

7.) Fig.6: With respect to an overall increase in T cells in the liver, please provide absolute cell numbers for CD69+, CD44hiCD62Llow, CD127+CD62L+ CD8+ T cells.
Response:

We added the data to (**Supplementary Fig. 3**). Consistent with their change in percentages, the absolute number of $CD69^+CD8^+$ T cells (**Supplementary Fig. 3a**), $CD25^+CD8^+$ T cells (**Supplementary Fig. 3b**), $CD44^{hi}CD62L^{low}CD8^+$ T cells (**Supplementary Fig. 3c**) and $Ki67^+CD8^+$ T cells (**Supplementary Fig. 3d**) increased dramatically in the liver of TIGIT-blockade group. The absolute number of liver central memory $CD8^+$ T cells ($CD127^+CD62L^+$) mildly decreased (**Supplementary Fig. 3e**).

Minor points:
1) Fig.5C: Please do not pre-gate on CD8+. Showing CD8 vs HBsAg-tetramer also on CD8- cells will help to evaluate the quality of the HBsAg-tetramer staining.
Response:

As advised by the reviewer, we have modified the gating strategy in the revised version (**Fig.5c, d**).

2) Fig.6G/6I: Please show negative controls (w/o PMA/Ionomycin stimulation) and do not pre-gate on CD8+.
Response:

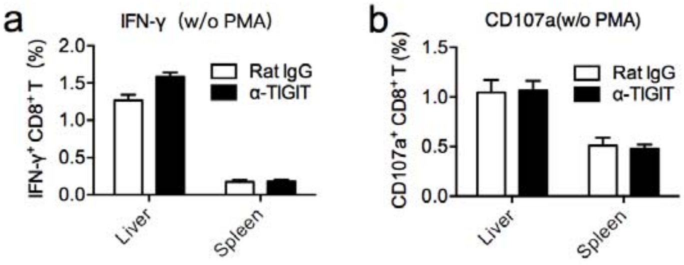

Legend: Expression of intracellular IFN-γ (**a**) and CD107a (**b**) by hepatic and splenic $CD8^+$ T cells of mice without stimulation of HBs-tg mice after TIGIT or rat IgG treated. 5 mice per group. Data are presented as the mean ±

SEM.

Thanks for the suggestion. The representative flow cytometric graphs without stimulation were shown in **Fig.6k.m** in the revised version. The statistical results are shown in upper figure. The flow cytometric graphs were not pre-gated on $CD8^+$ cells as advised by you, and the statistics showed the frequency of IFN-γ^+/$CD107a^+$ $CD8^+$ T cells among total $CD3^+$ T cells.

3.) Page 7, line 25: Please be precise in applying the concept of exhaustion and tolerance. These are two different immunological situations.

Response:

Thanks for the suggestion. To avoid the conceptual confusion, the sentence in the previous version "These results suggest that TIGIT blockade accompanies the functional switch of hepatic $CD8^+$ T cells from functional exhaustion to competence in HBs-tg mice" was changed as "These results suggest that TIGIT blockade accompanies the functional restoration of hepatic $CD8^+$ T cells in HBs-tg mice." These changes were highlighted in the revised version of manuscript.

Sincerely Yours,

Zhigang Tian, M.D., Ph.D.

Director & Professor, Institute of Immunology, School of Life Sciences and Medical center
Director, Key Laboratory of Innate Immunity and Chronic disease, Chinese Academy of Sciences
President, Medical Center
University of Science & Technology of China
Hefei, Anhui 230027, China
Tel: 086-551-360-0845
Fax: 086-551-360-6783
E-mail: tzg@ustc.edu.cn

Reviewers' Comments:

Reviewer #1:
Remarks to the Author:
The authors have extensively revised the ms and no new issue.

Reviewer #2:
Remarks to the Author:
In the revised manuscript, the authors supplemented more data to support their assumption. However, the major concern is whether HCC developed in the mice as suggested by the authors. From the perspective of pathology, Fig.2e, Fig.3d, Fig. 8j are not convincing for the successful establishment of tumors in the mice, even some cells showed positivity for markers such as AFP. Also, for supplementary Fig 2d, most red colored signals in the figure didn’t match with the staining of DAPI. This raises the concern that these are not real signals for CD8+ cells but background noise.

Reviewer #3:
Remarks to the Author:
The authors have addressed all may suggestions. Also, the quality of tertramer staining displayed now is convining.

Response to the Reviewers' comments point-by-point

Reviewer #1

The authors have extensively revised the ms and no new issue.

Response:

Thank you very much for your constructive suggestions, which have helped us to greatly improve the manuscript.

Reviewer #2:

1. In the revised manuscript, the authors supplemented more data to support their assumption. However, the major concern is whether HCC developed in the mice as suggested by the authors. From the perspective of pathology, Fig.2e, Fig.3d, Fig. 8j are not convincing for the successful establishment of tumors in the mice, even some cells showed positivity for markers such as AFP. (Previous revision question：In figure 2d, the structure of tumor in a-TIGIT+vaccine group was hardly to tell from other benign hyperplastic nodules. The authors need to provide more evidence to confirm the cancerous features of this entity (such as AFP staining, genetic analysis etc.) This also applies to Fig 3C.)

Response:

Thank you for your valuable comments. In the revised version, we provided experimental data to convincingly verify that the mice developed HCC. We utilized a new polyclonal antibody (ProteinTech, catalog#14550-I-AP) for α-fetoprotein (AFP) staining instead of the previous anti-AFP antibody we used (Agilent Technologies, DAKO, catalog#IS500). As shown in **Fig.2e and Fig.3d** of the revised version, hepatocytes in tumoral area of HBsAg vaccinated α-TIGIT-treated or *Tigit*$^{-/-}$ HBs-tg mice are largely AFP positive. Q-PCR analysis consistently showed that liver tissues of these mice had more *Afp* gene expression than control group (**Fig. 2f and Fig. 3e**). Moreover, inside the tumor (AFP positive region), the cells are dysplastic based on their morphological feature and had pleomorphic nuclei. Additionally, blood vessels existed at the boundary zone. This is consistent with the viewpoint that early HCC of the distinctly nodular type and progressed-HCC appear hypervascular because of neovascularization (Manuel Schlageter et al, World Journal of Gastroenterology, 2014). Besides, trabecular pattern can also be seen in **Fig.3d**. For **Fig8j**, we provided a more representative H-E staining figure. As shown in this figure, the cells in the tumoral area display increased nuclear/cytoplasmatic ratio, nuclear hyperchromasia, irregular nuclear and prominent nucleoli. For the histological features described above, we conclude that HCC occurred in these mice.

2. Also, for supplementary Fig 2d, most red colored signals in the figure didn't match with the staining of DAPI. This raises the concern that these are not real signals for CD8+ cells but background noise. (Previous revision question：In figure 1c, infiltration of leukocytes in the liver tissue was shown. But whether these cells are CD8+ T cells or not was not examined.)

Response:

Thank you for your reminder. To improve the quality of this figure**,** we adjusted the time of primary antibody incubation and washing times, when we repeated the immunofluorescence staining experiments. We presented the data to in **Supplementary Fig. 2d** of the revised version, in which we can see the infiltrated cells contained CD8 positive cells. There were also CD8 negative cells among the infiltrated cells, and they may be monocytes and neutrophils recruited to liver. For the CD8 positive cells, the DAPI was surrounded by CD8 staining.

Reviewer #3:

The authors have addressed all may suggestions. Also, the quality of tertramer staining displayed now is convining.

Thank you very much. We really appreciate your careful review and valuable comments.

With my best regards,

Zhigang Tian, Ph.D., M.D.
Director & Professor, Institute of Immunology,
School of Life Sciences and Medical center,
University of Science & Technology of China
Hefei 230027, China
E-mail: tzg@ustc.edu.cn
Tel: 86-551-6360-0845;
Fax: 86-551-360-6783.

Reviewers' Comments:

Reviewer #2:
Remarks to the Author:
The authors have addressed the concerns.

Response to the Reviewers' comments point-by-point

Reviewer #2

The authors have addressed the concerns.

Response:

Thank you for the insightful suggestions. We really appreciate your comments which greatly improve the quality of the manuscript.
